# Supplementary material for: Phosphorus-Derived Isatin Hydrazones: Synthesis, Structure, Thromboelastography, Antiplatelet, and Anticoagulation Activity Evaluation
Source: Int J Mol Sci. 2025 Jun 26;26(13):6147. doi: 10.3390/ijms26136147 (PMC12249729; doi:10.3390/ijms26136147)
Supplement: Supplementary file 1 [file ijms-26-06147-s001.zip › ijms-3705073-supplementary.pdf]

SUPPLEMENTARY MATERIALS

Phosphorus-Derived Isatin Hydrazones: Synthesis, Structure, Thromboelastography, Antiplatelet, and Anticoagulation Activity Evaluation

Aleksandr V. Samorodov [1,*], Wang Yi [2], Dmitry A. Kudlay [3,4], Elena A. Smolyarchuk [3], Alexey B. Dobrynin [5,6], Ayrat R. Khamatgalimov [5], Karina Shchebneva [5], Marina Kadomtseva [7], Dilbar Komunarova [8], Anna G. Strelnik [5] and Andrei V. Bogdanov [5,*]

[1] Department of Pharmacology, Bashkir State Medical University, Lenin St. 3, Ufa 450008, Russia
[2] School of Pharmacy, Hangzhou Normal University, Yuhangtan, 2318, Hangzhou 310030, China; yi.wang1122@hznu.edu.cn
[3] Department of Pharmacology, Sechenov First Moscow State Medical University (Sechenov University), Bolshaya Pirogovskaya St. 2, Building 4, Moscow 119435, Russia; kudlay_d_a@staff.sechenov.ru (D.A.K.); smolyarchuk@mail.ru (E.A.S.)
[4] Institute of Immunology FMBA of Russia, Kashirskoye Highway, Building 24, Moscow 115522, Russia
[5] Arbuzov Institute of Organic and Physical Chemistry, FRC Kazan Scientific Center, Russian Academy of Sciences, Akad. Arbuzov St. 8, Kazan 420088, Russia; aldo@iopc.ru (A.B.D.); khamatgalimov@gmail.com (A.R.K.); shchebnevak@mail.ru (K.S.); nikanna@iopc.com (A.G.S.)
[6] Department for design and techiques of electronic tools manufacturing, Institute for radio-electronics and telecommunications, Kazan National Research Technical University Named After A. N. Tupolev-KAI, Karl Marx St. 10, Kazan 420111, Russia
[7] A.M. Butlerov Institute of Chemistry, Kazan Federal University, Kremlevskaya St. 29, Kazan 420008, Russia; kozyreva_marina_@mail.ru
[8] Department of Organic Chemistry, Kazan National Research Technological University, Sibirsky Trakt 12, Kazan 420029, Russia; dil6744@mail.ru
* Correspondence: avsamorodov@gmail.com (A.V.S.); abogdanov@inbox.ru (A.V.B.)

Contents

X-Ray data	2
Copies of NMR spectra	3-34
Copies of IR spectra	35-46
Copies of mass-spectra	47-58

X-Ray data

Table S1. The detailed x-ray data for the compounds **3e** and **5e**

**Compound**	**3e**	**5e**
Chemical formula	$C_{22}H_{17}FN_3O_3P$	$C_{20}H_{21}ClFN_4O_4P$
M_r	421.35	466.83
Crystal system	monoclinic	monoclinic
Space group	*C2/c*	$P2_1/c$
Temperature (K)	150	100
a, *b*, *c* (Å)	22.1377(14)	12.9091(4)
	17.7538(12)	14.8559(5)
	11.3218(7)	22.3242(7)
	117.824(2)	98.4930(10)
V (Å^3)	3935.3(4)	4234.3(2)
Z	8	8
D(calc) [g/cm^3]	1.422	1.465
Radiation type	Mo *Kα*	
μ (mm^{-1})	0.179	0.301
Crystal size (mm)	0.15 × 0.11 × 0.09	0.15 × 0.12 × 0.08
Diffractometer	Bruker D8 QUEST	Bruker D8 QUEST
T_{min}, T_{max}	0.6226, 0.7461	0.7183 , 0.7473
No. of measured reflections	60651	361378
No. of independent reflections	6019	19821
No. of observed [I > 2s(I)] reflections	5039	17309
R_{int}	0.0579	0.037
R_1/ $wR(F^2)$, $[I>2\sigma]$	0.0389 / 0.1069	0.0376 / 0.1038
R_1/ $wR(F^2)$, (all data)	0.0470 / 0.1151	0.0438 / 0.1069
GOOF	1.068	1.067
No. of reflections	6019	19821
No. of parameters	271	563
No. of restraints	0	0
Dρ_{max}, Dρ_{min} (e Å^{-3})	0.51/-0.33	0.98/-0.77
**CCDC no**	XXXXXXX	ZZZZZZZ

Copies of NMR spectra

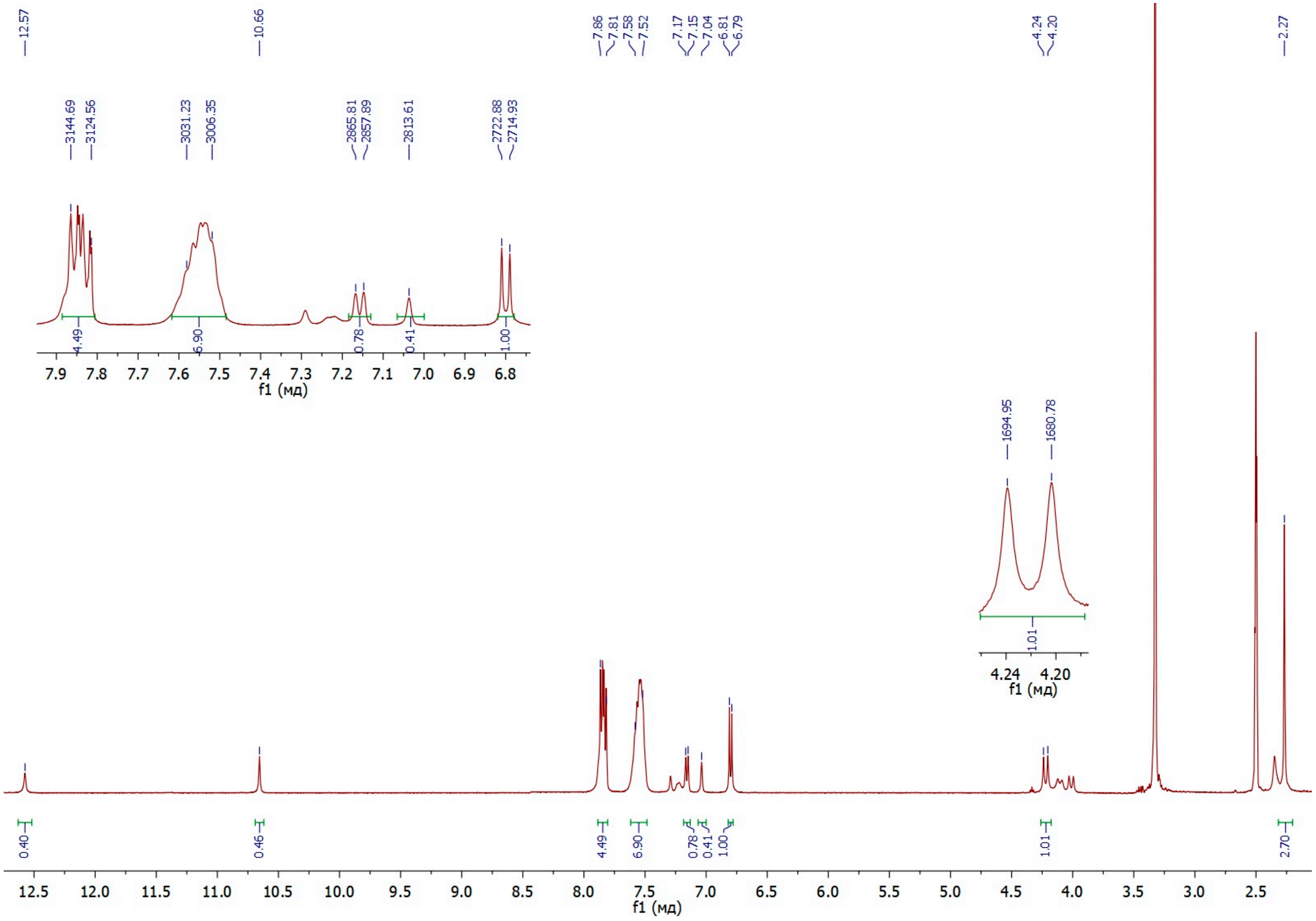

Figure S1. ^{1}H NMR spectrum of compound **3a** (400 MHz, DMSO-d_6)

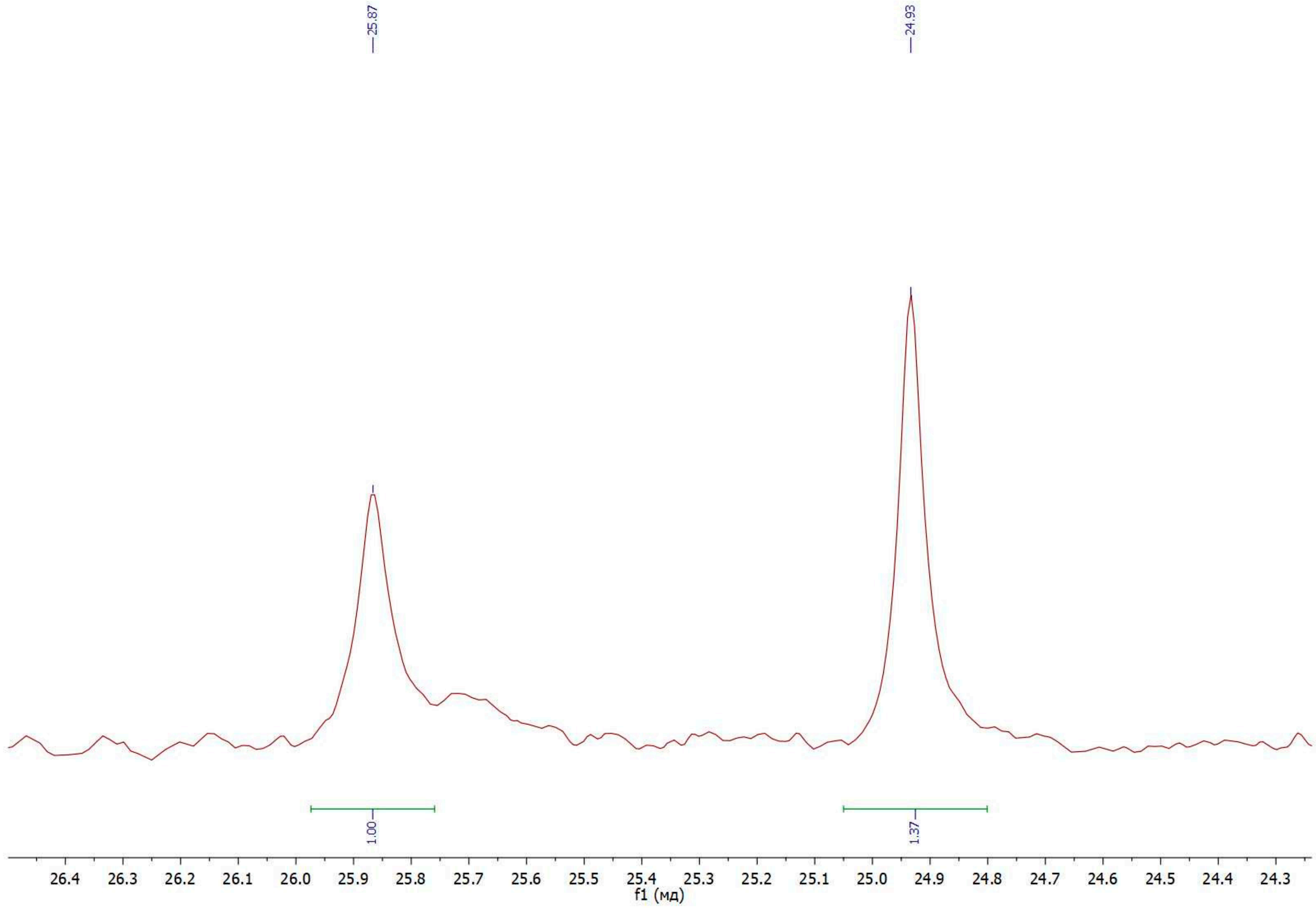

Figure S2. ^{31}P NMR spectrum of compound **3a** (162 MHz, DMSO-d_6)

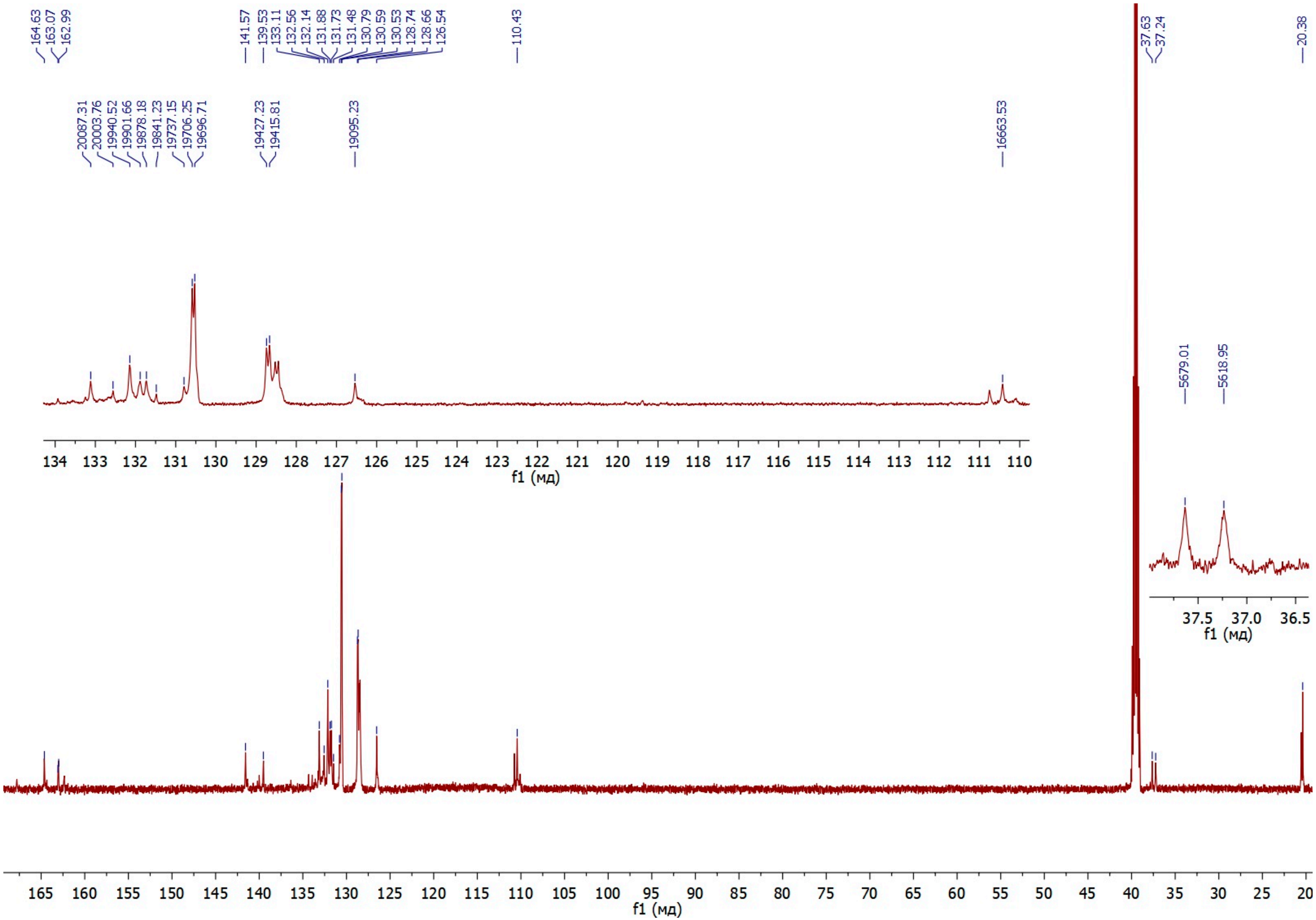

Figure S3. ^{13}C-{^{1}H} NMR spectrum of compound **3a** (151 MHz, DMSO-d_6)

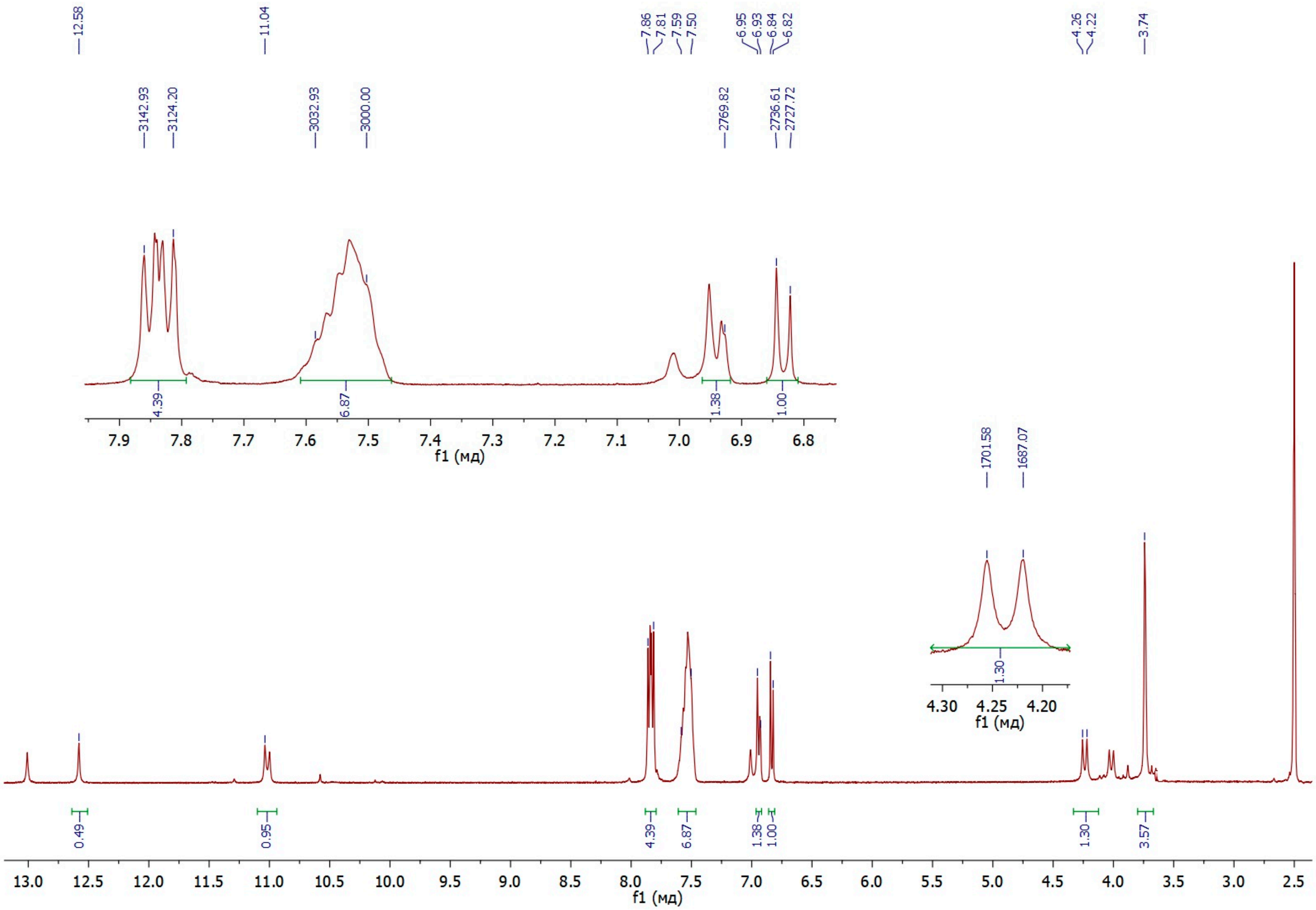

Figure S4. ^{1}H NMR spectrum of compound **3b** (600 MHz, DMSO-d_6)

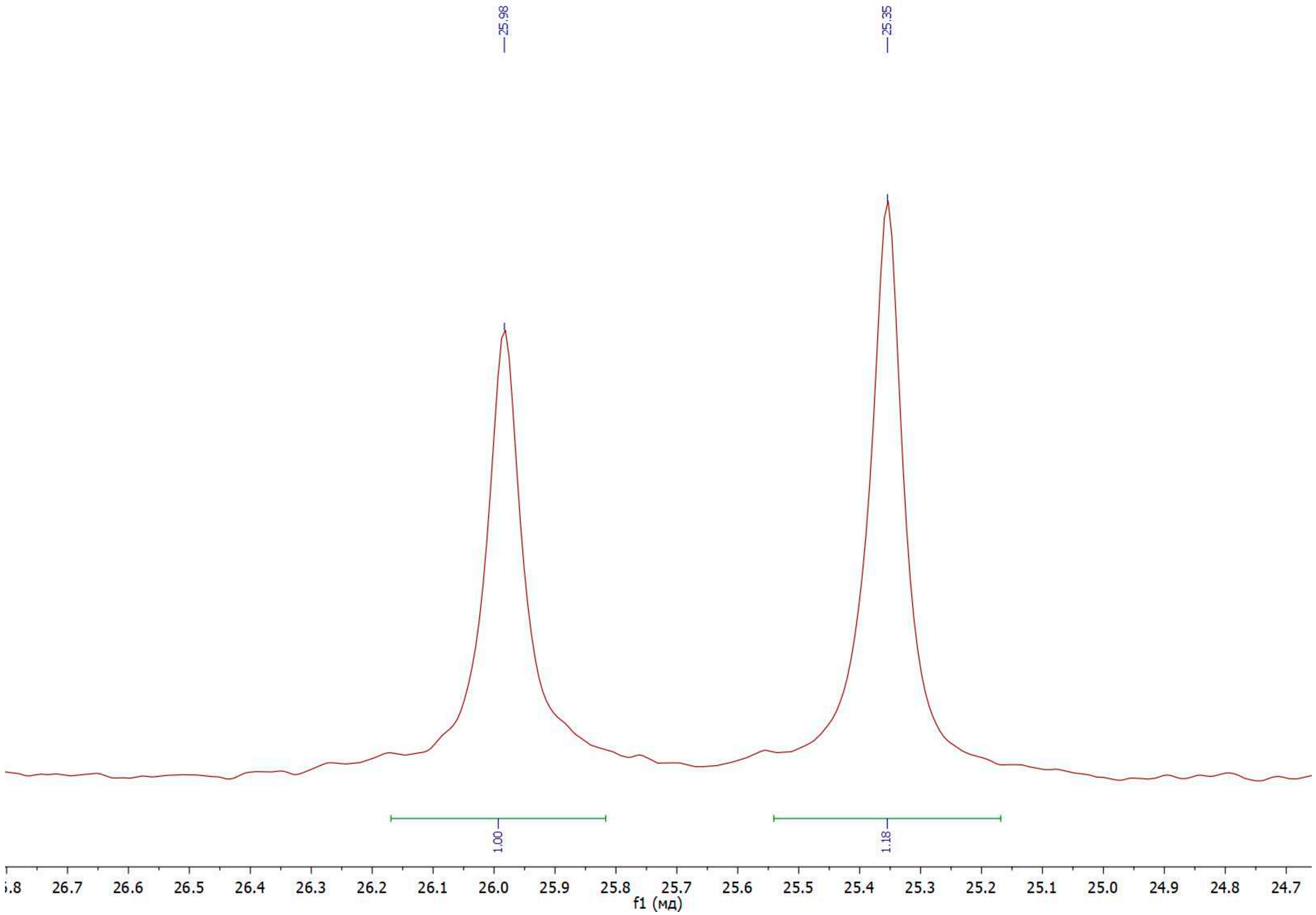

Figure S5. ^{31}P NMR spectrum of compound **3b** (243 MHz, DMSO-d_6)

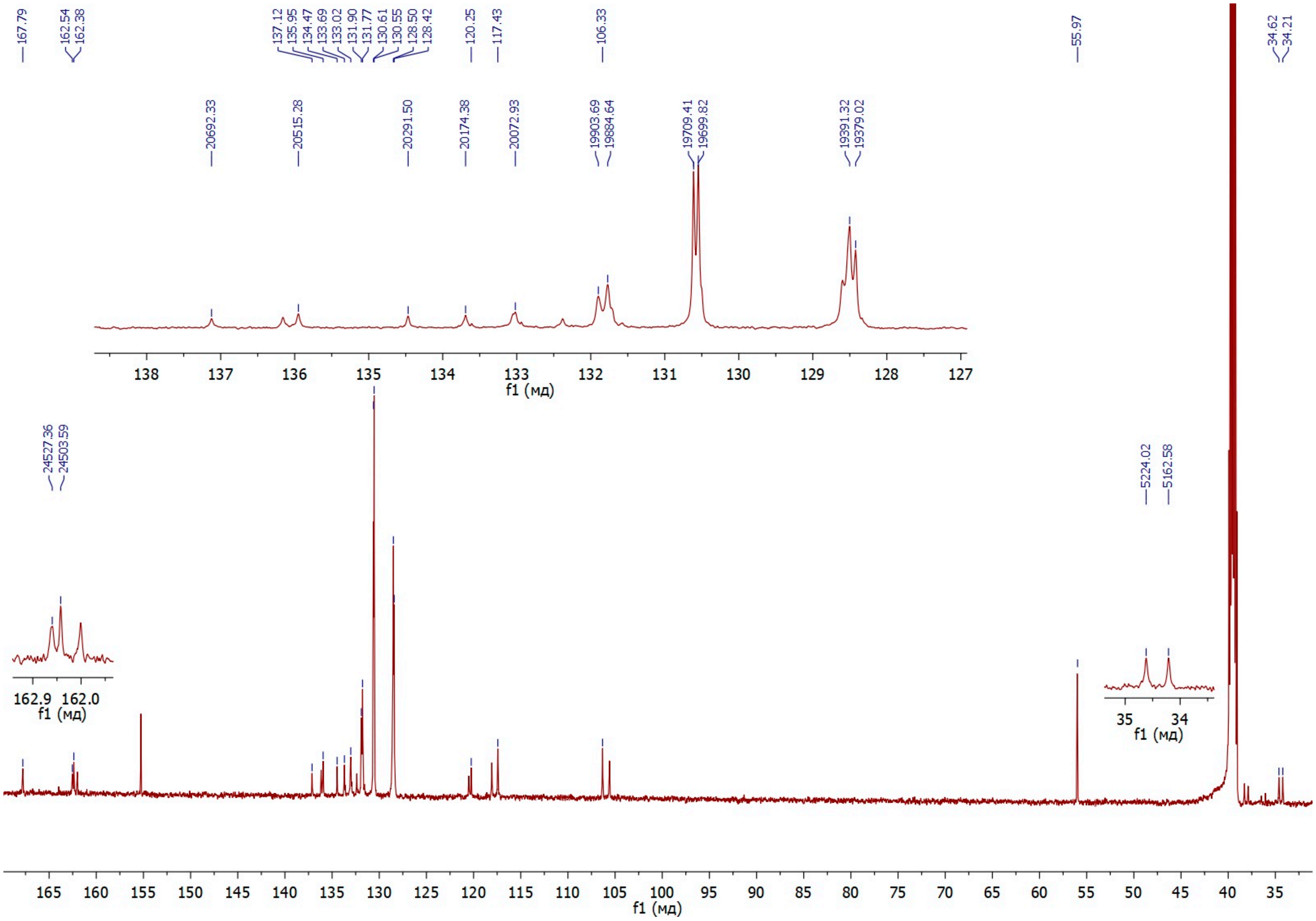

Figure S6. ^{13}C-{^{1}H} NMR spectrum of compound **3b** (151 MHz, DMSO-d_6)

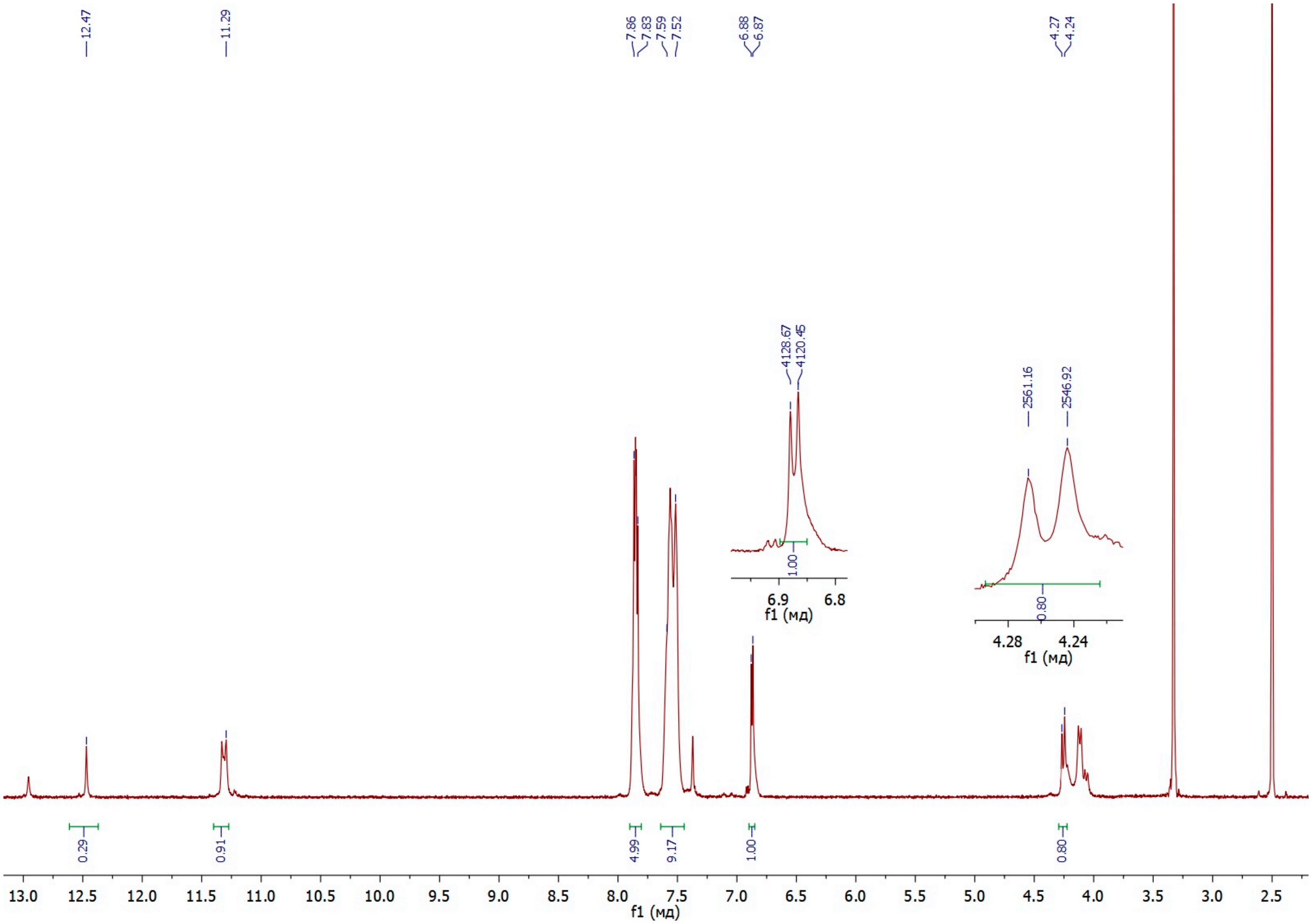

Figure S7. ^{1}H NMR spectrum of compound **3c** (400 MHz, DMSO-d_6)

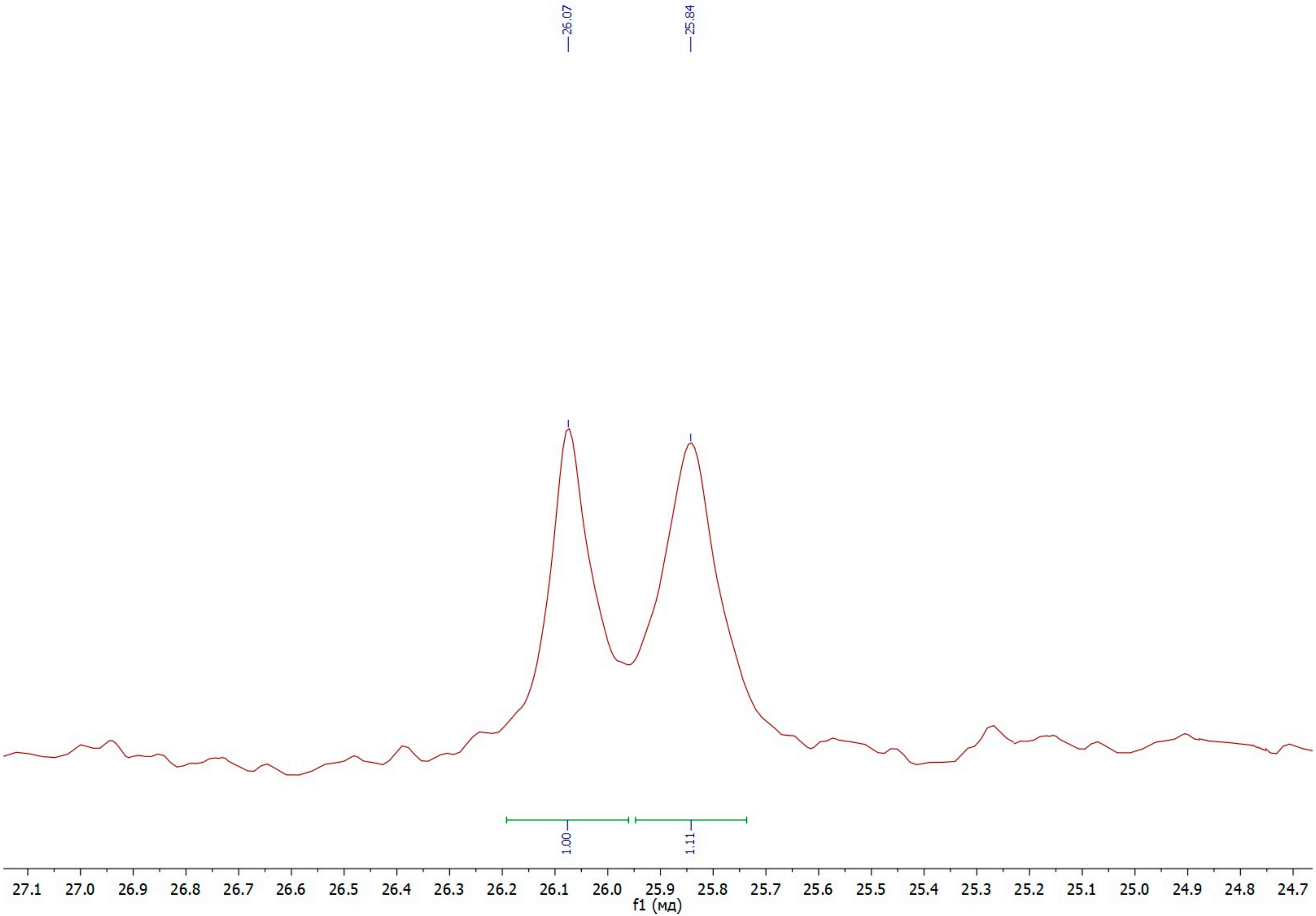

Figure S8. ^{31}P NMR spectrum of compound **3c** (243 MHz, DMSO-d_6)

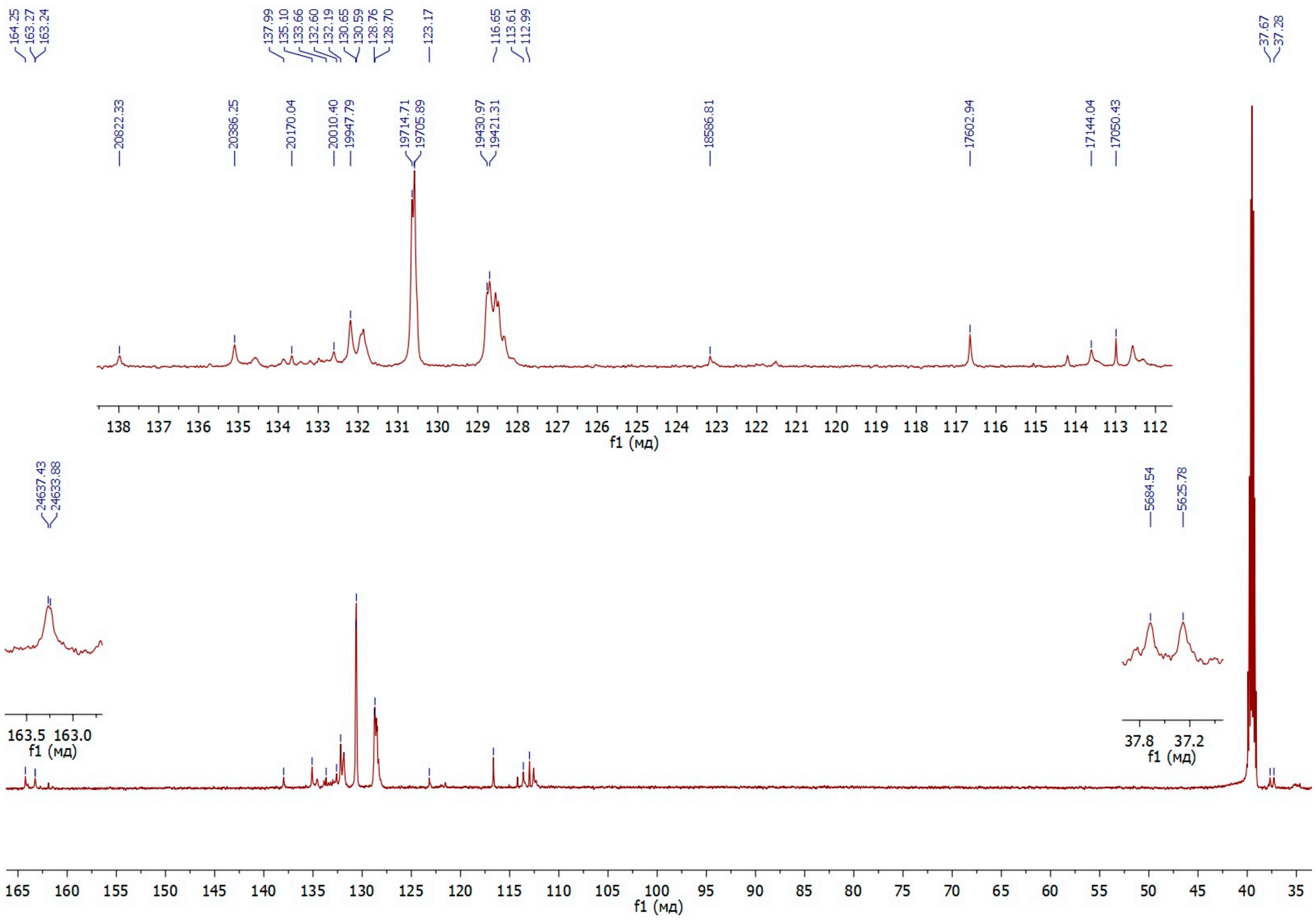

Figure S9. ^{13}C-{^{1}H} NMR spectrum of compound **3c** (151 MHz, DMSO-d_6)

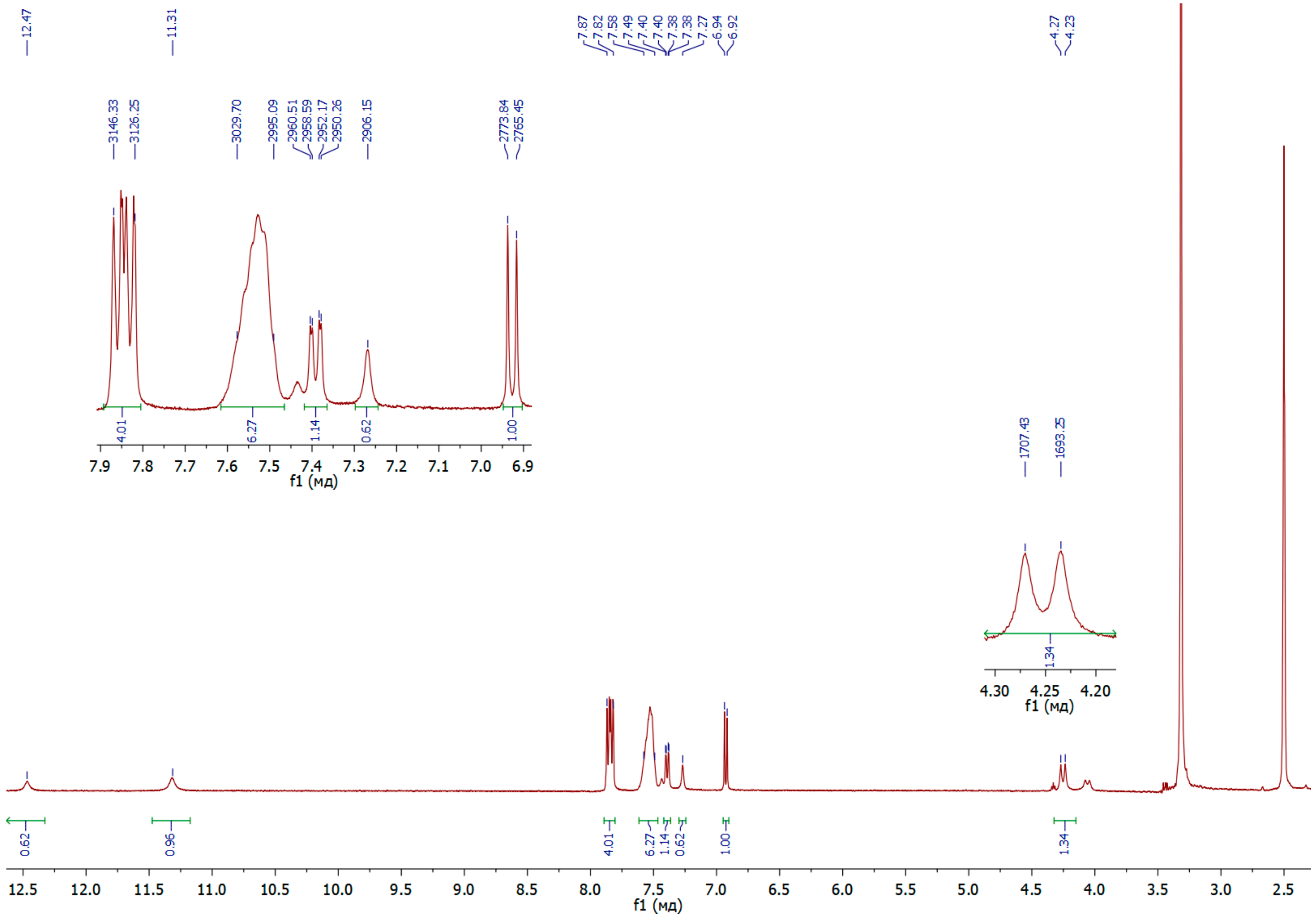

Figure S10. ^{1}H NMR spectrum of compound **3d** (600 MHz, DMSO-d_6)

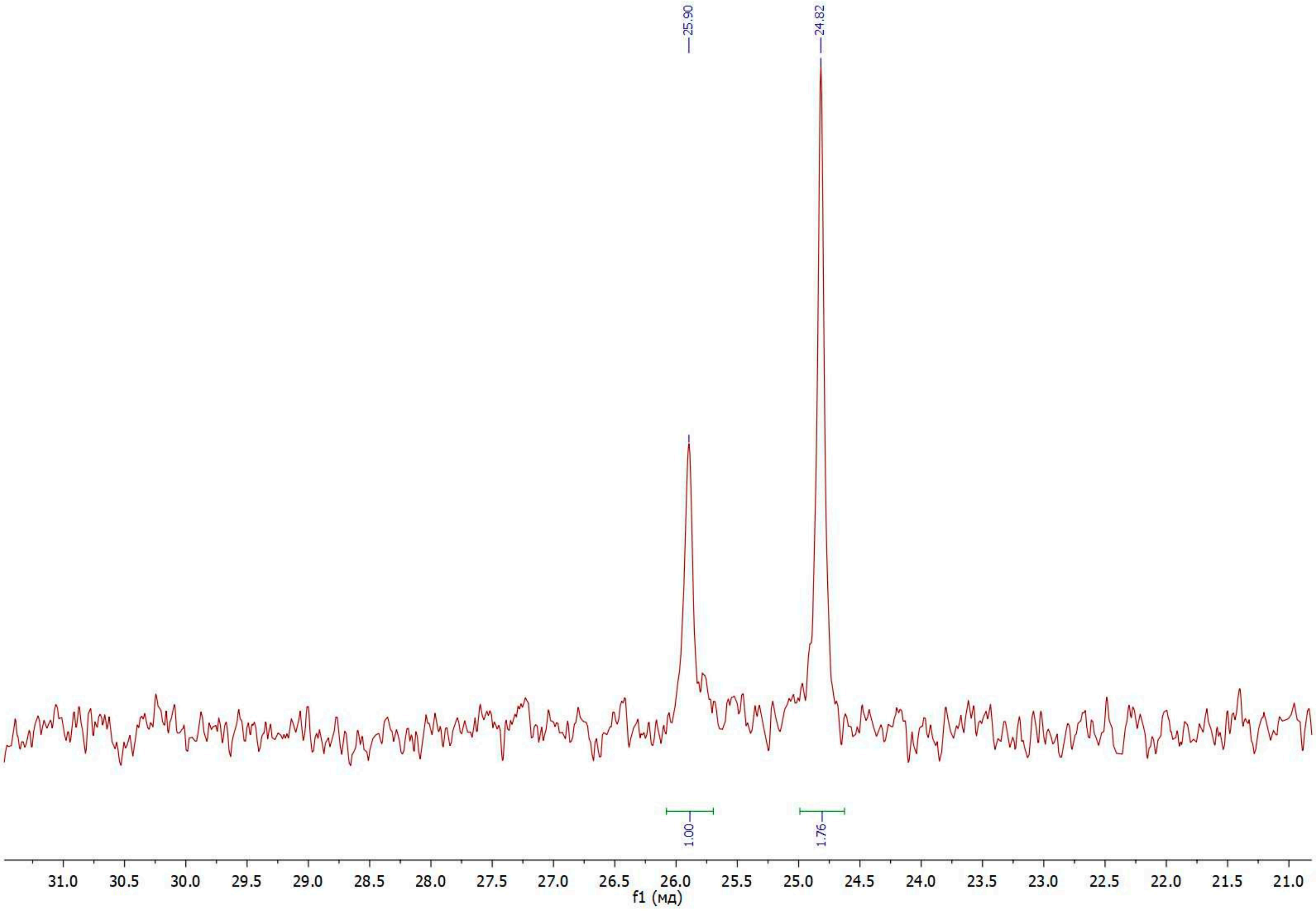

Figure S11. ^{31}P NMR spectrum of compound **3d** (243 MHz, DMSO-d_6)

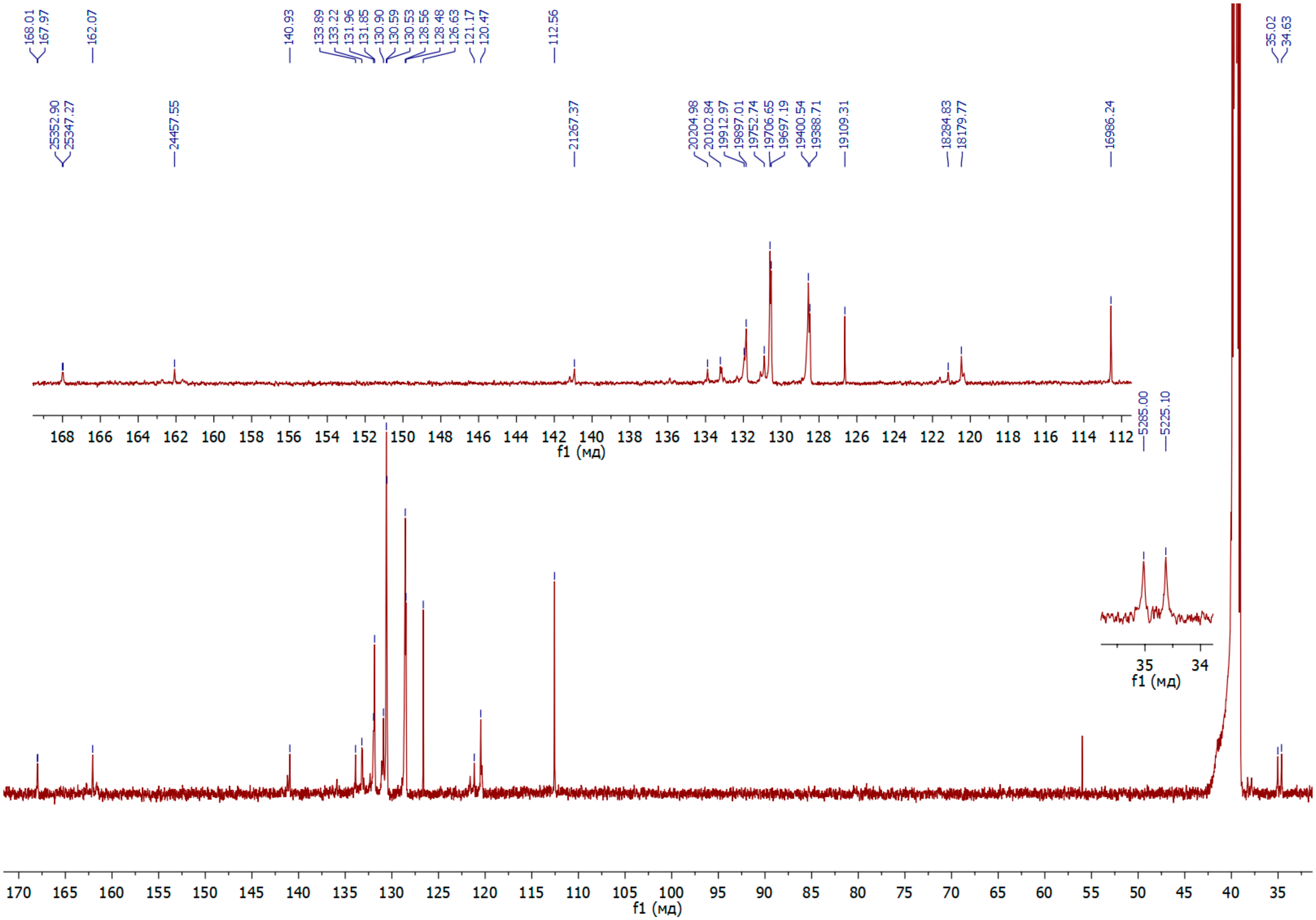

Figure S12. ^{13}C-{^1H} NMR spectrum of compound **3d** (151 MHz, DMSO-d_6)

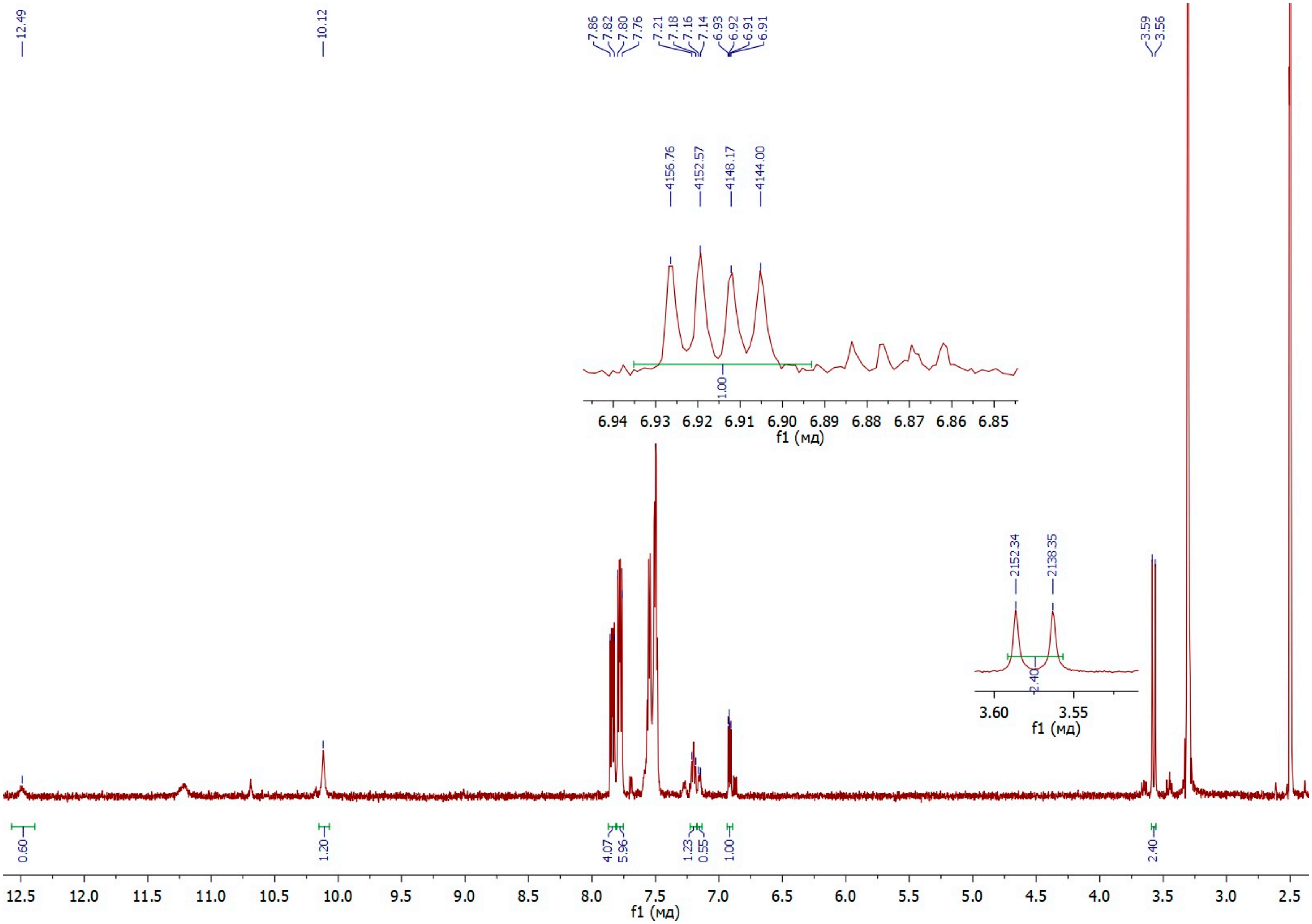

Figure S13. ^{1}H NMR spectrum of compound **3e** (600 MHz, DMSO-d_6)

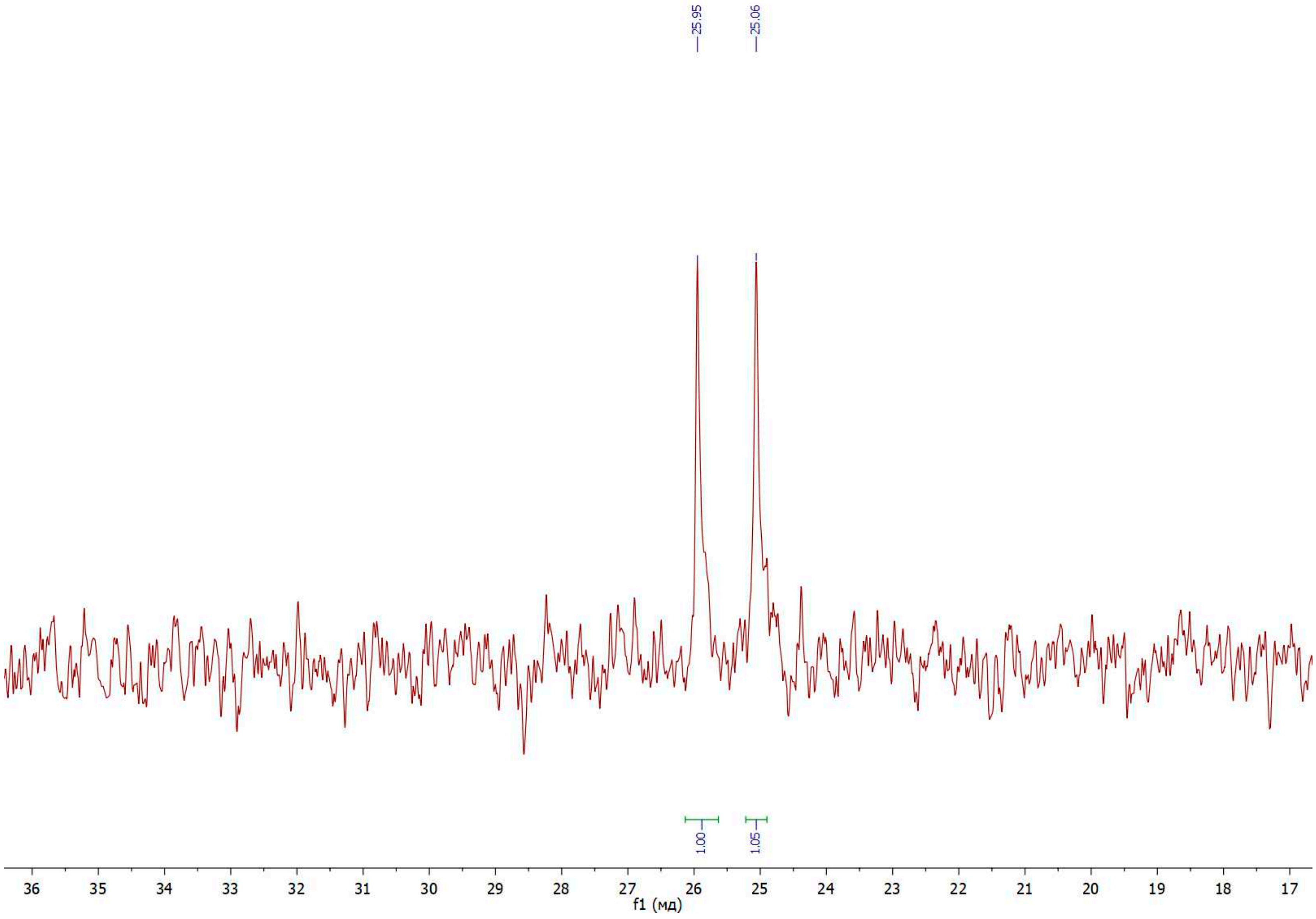

Figure S14. ^{31}P NMR spectrum of compound **3e** (243 MHz, DMSO-d_6)

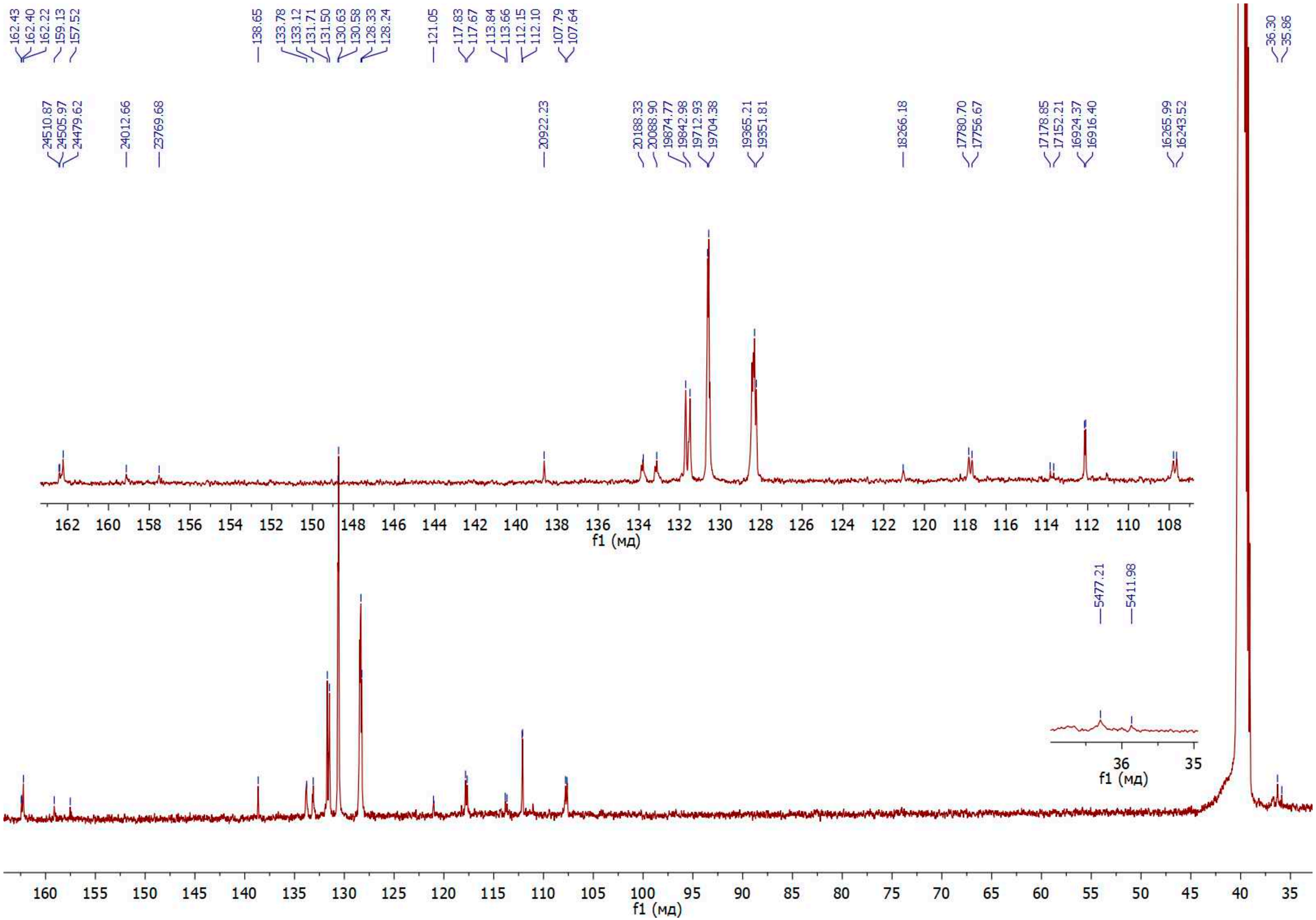

Figure S15. ${}^{13}C$-{${}^{1}H$} NMR spectrum of compound **3e** (151 MHz, DMSO-d_6)

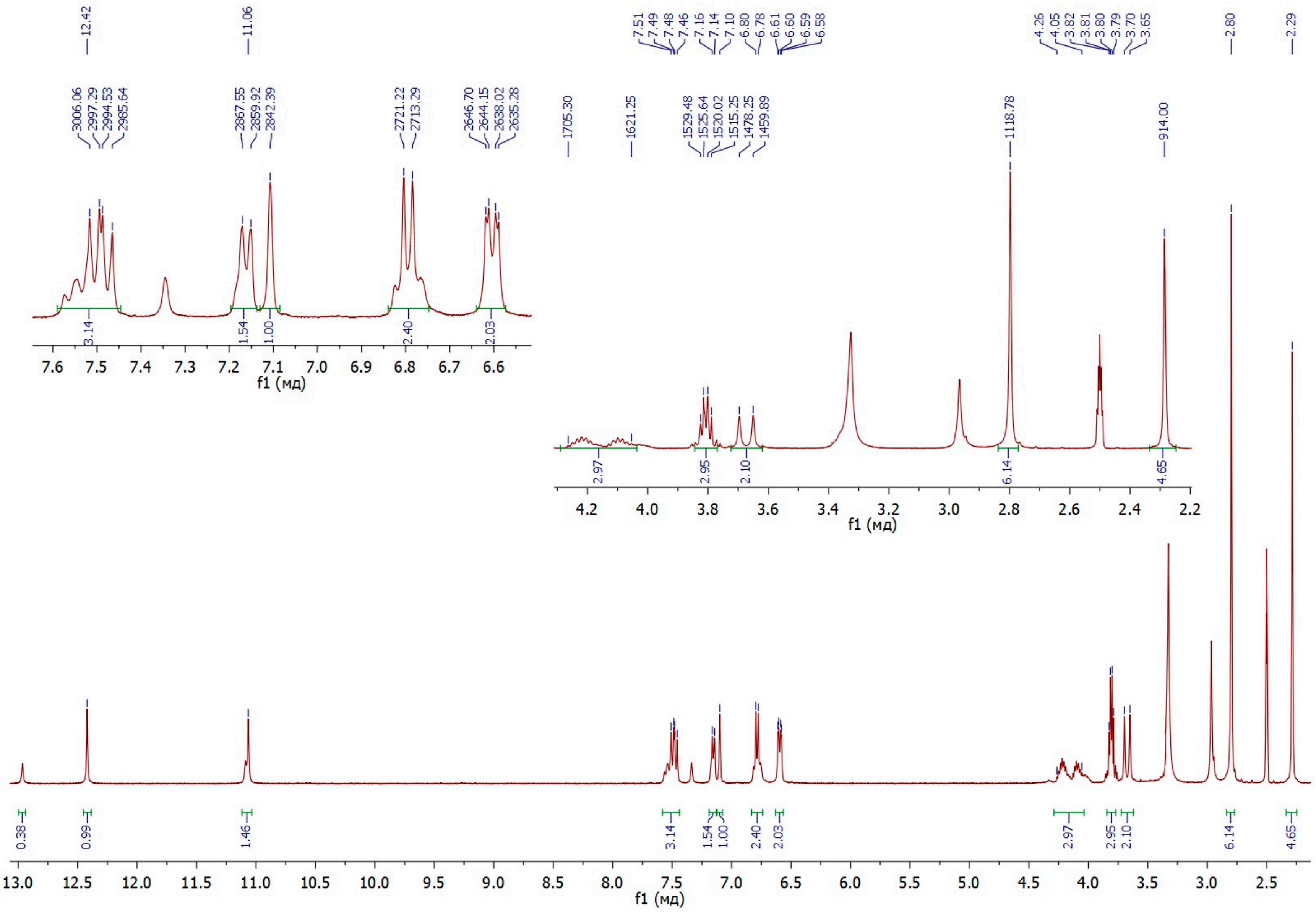

Figure S16. ^{1}H NMR spectrum of compound **5a** (500 MHz, DMSO-d_6)

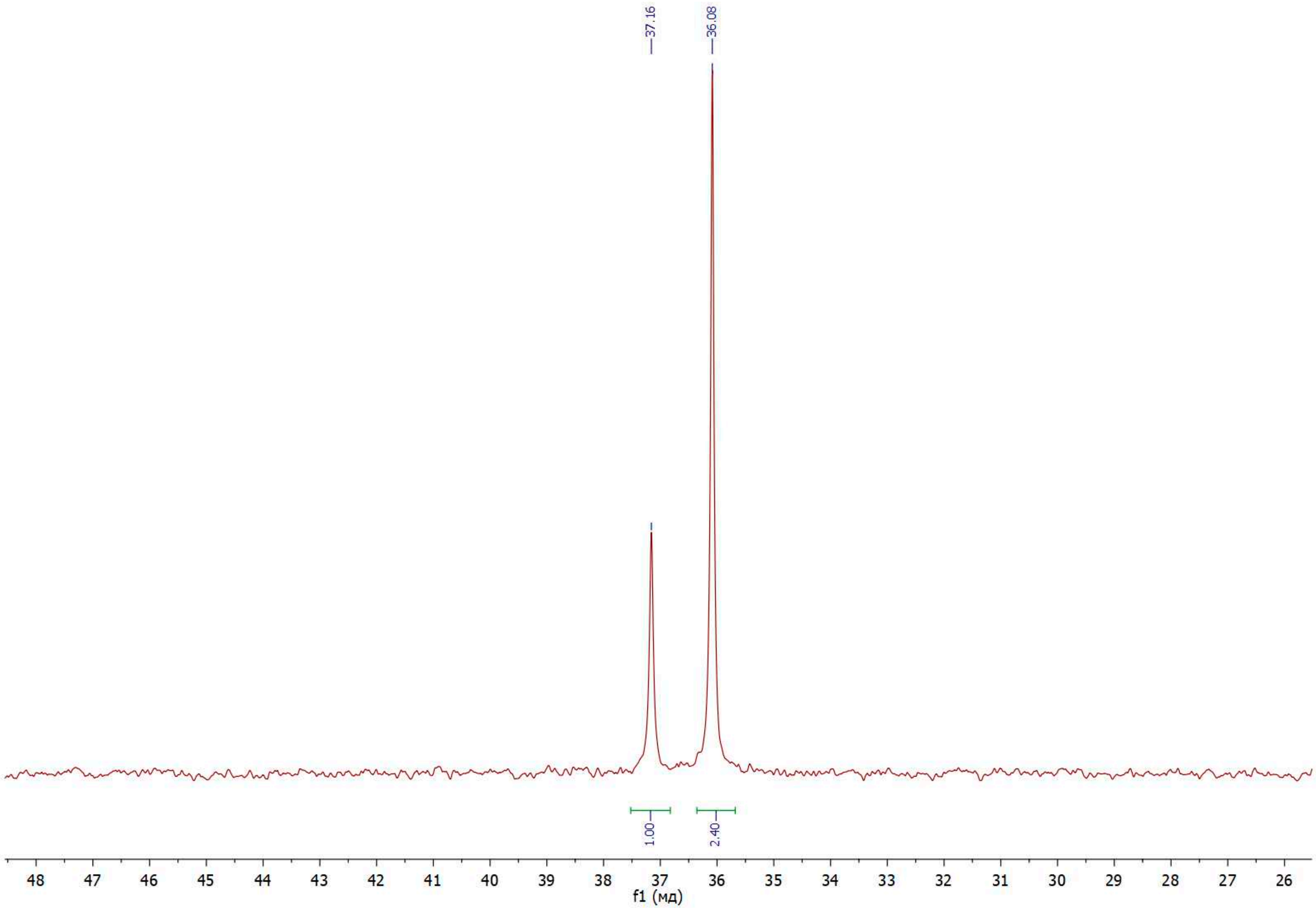

Figure S17. ^{31}P NMR spectrum of compound **5a** (162 MHz, DMSO-d_6)

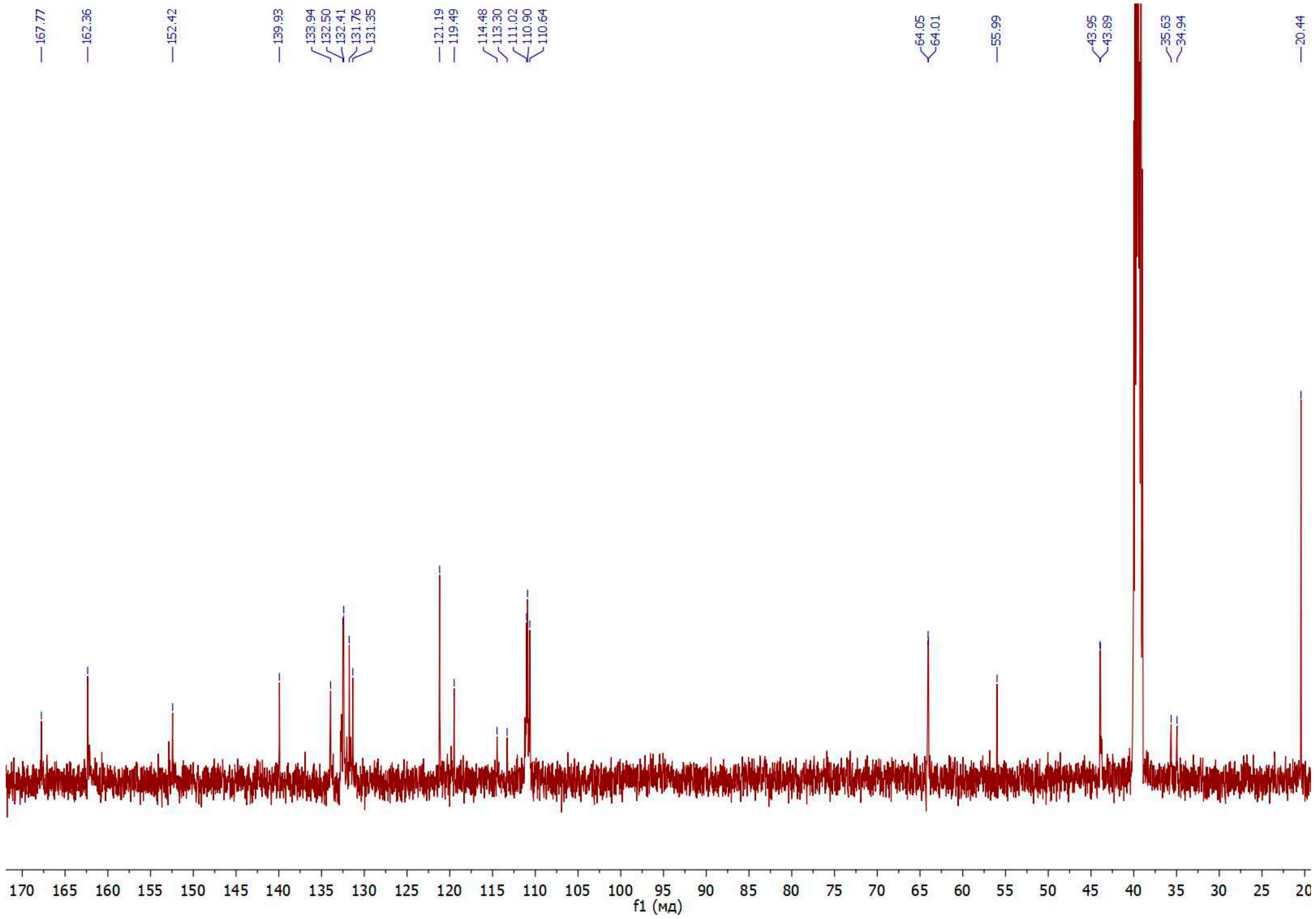

Figure S18. ^{13}C-{^{1}H} NMR spectrum of compound **5a** (126 MHz, DMSO-d_6)

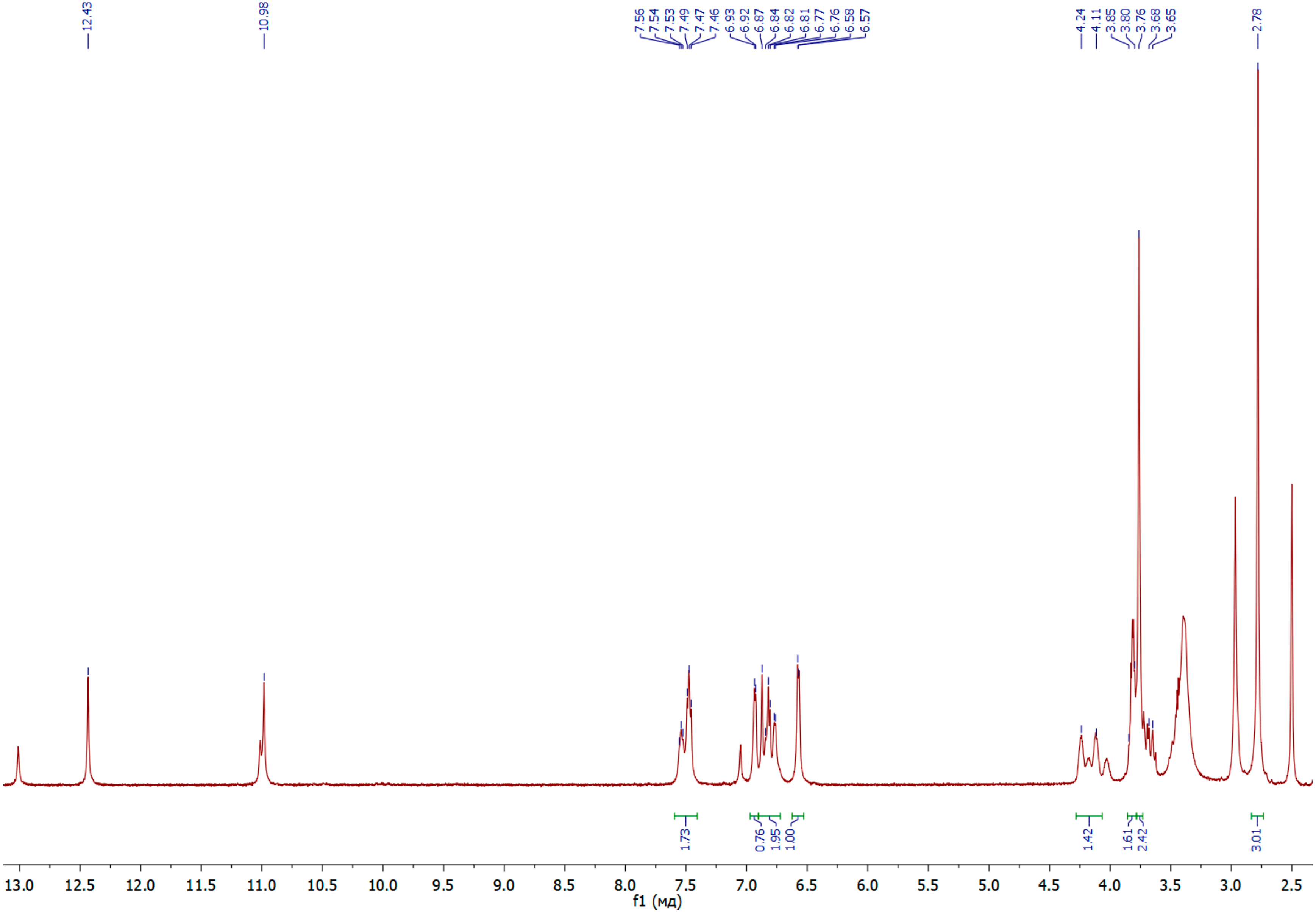

Figure S19. ^{1}H NMR spectrum of compound **5b** (500 MHz, DMSO-d_6)

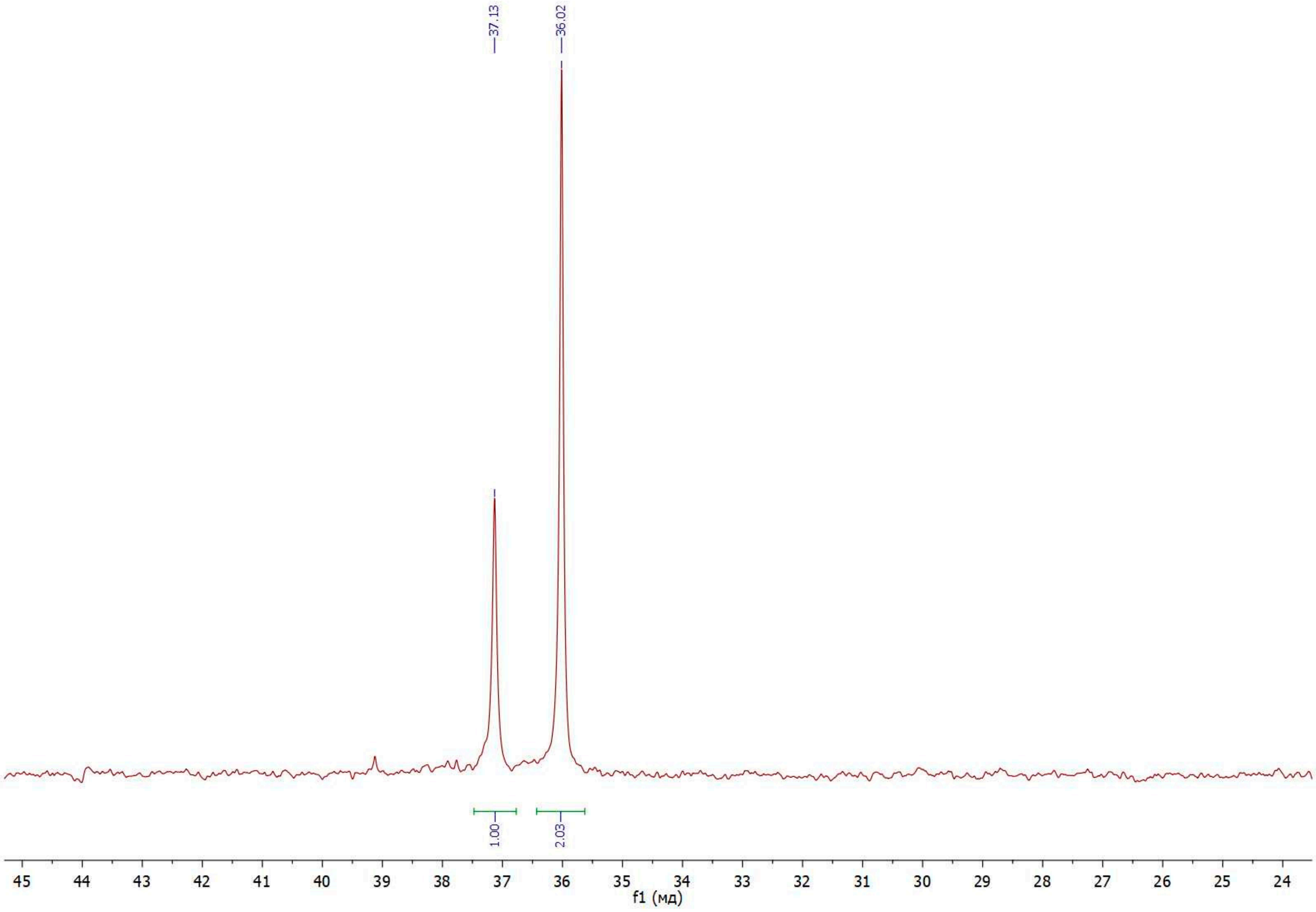

Figure S20. ^{31}P NMR spectrum of compound **5b** (162 MHz, DMSO-d_6)

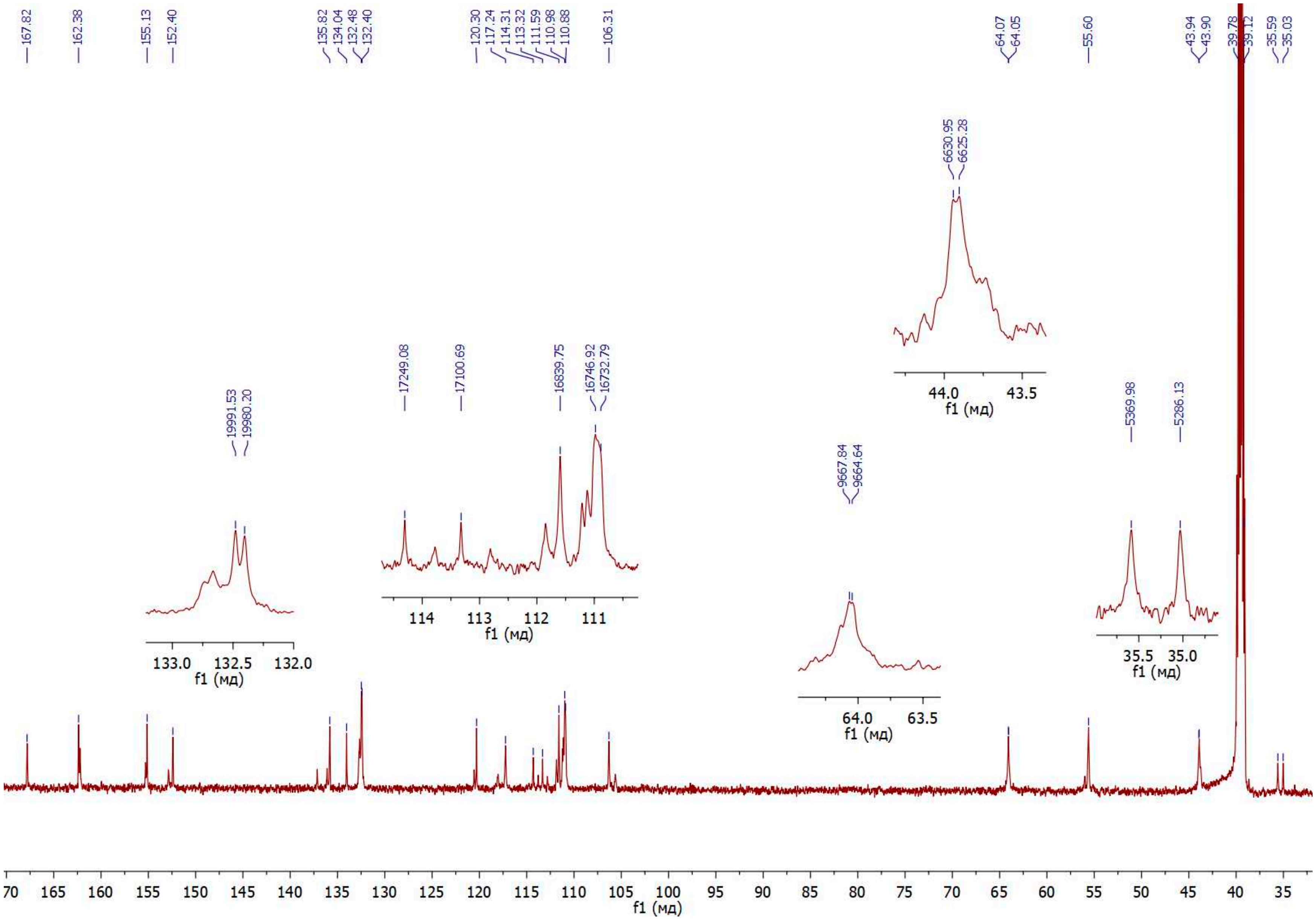

Figure S21. ^{13}C-{^{1}H} NMR spectrum of compound **5b** (151 MHz, DMSO-d_6)

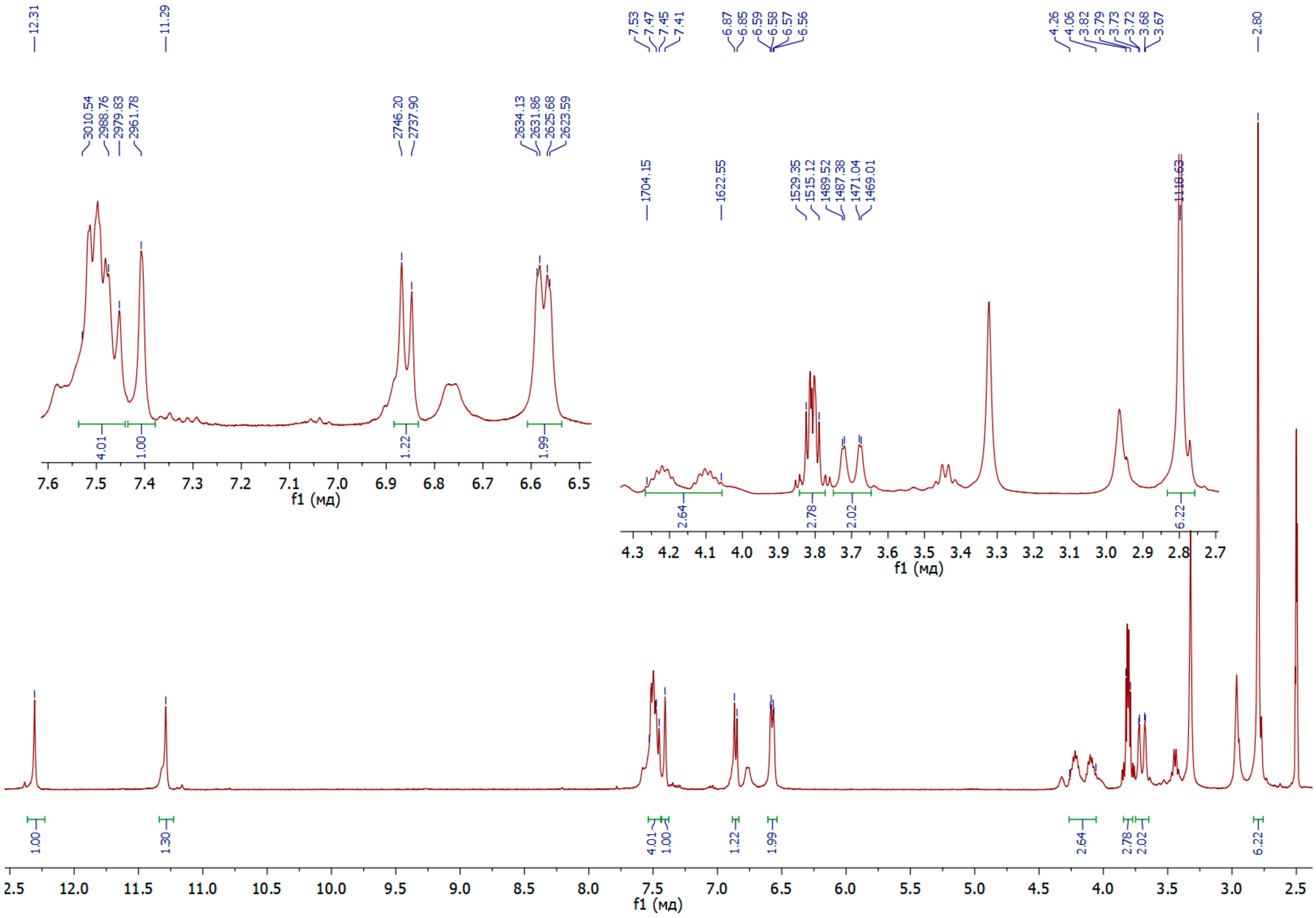

Figure S22. ^1H NMR spectrum of compound **5c** (400 MHz, DMSO-d_6)

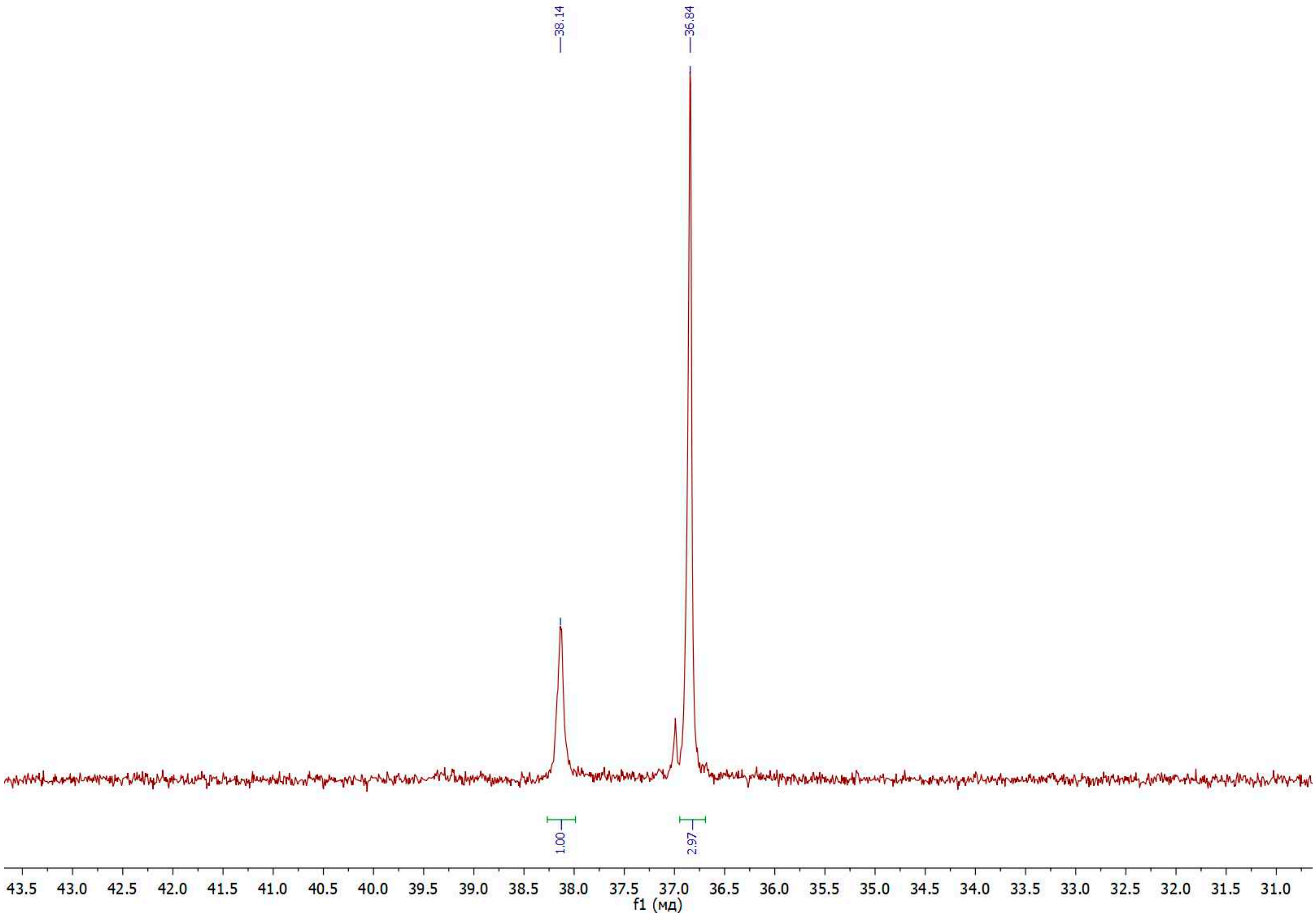

Figure S23. ^{31}P NMR spectrum of compound **5c** (243 MHz, DMSO-d_6)

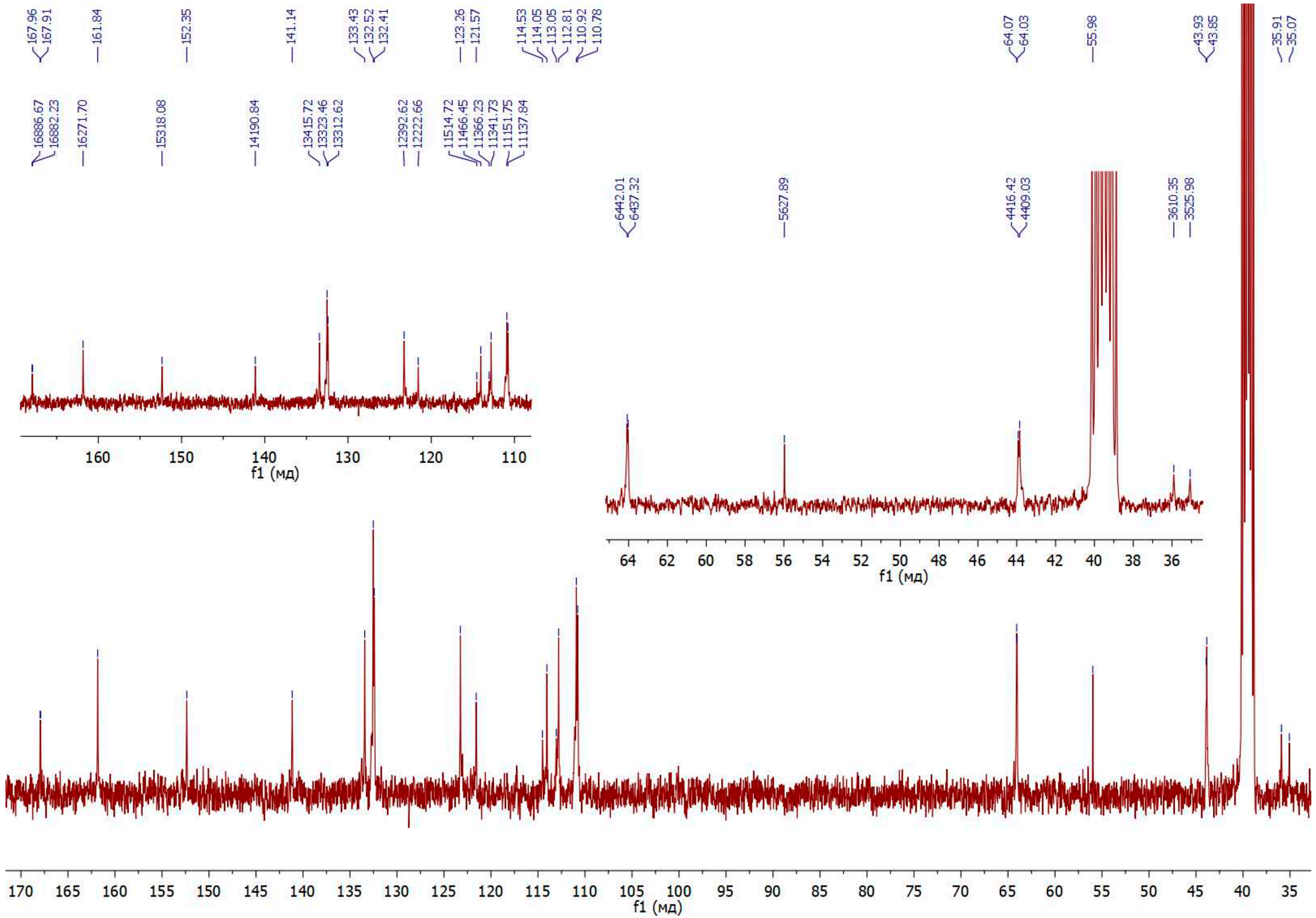

Figure S24. ^{13}C-{^{1}H} NMR spectrum of compound **5c** (101 MHz, DMSO-d_6)

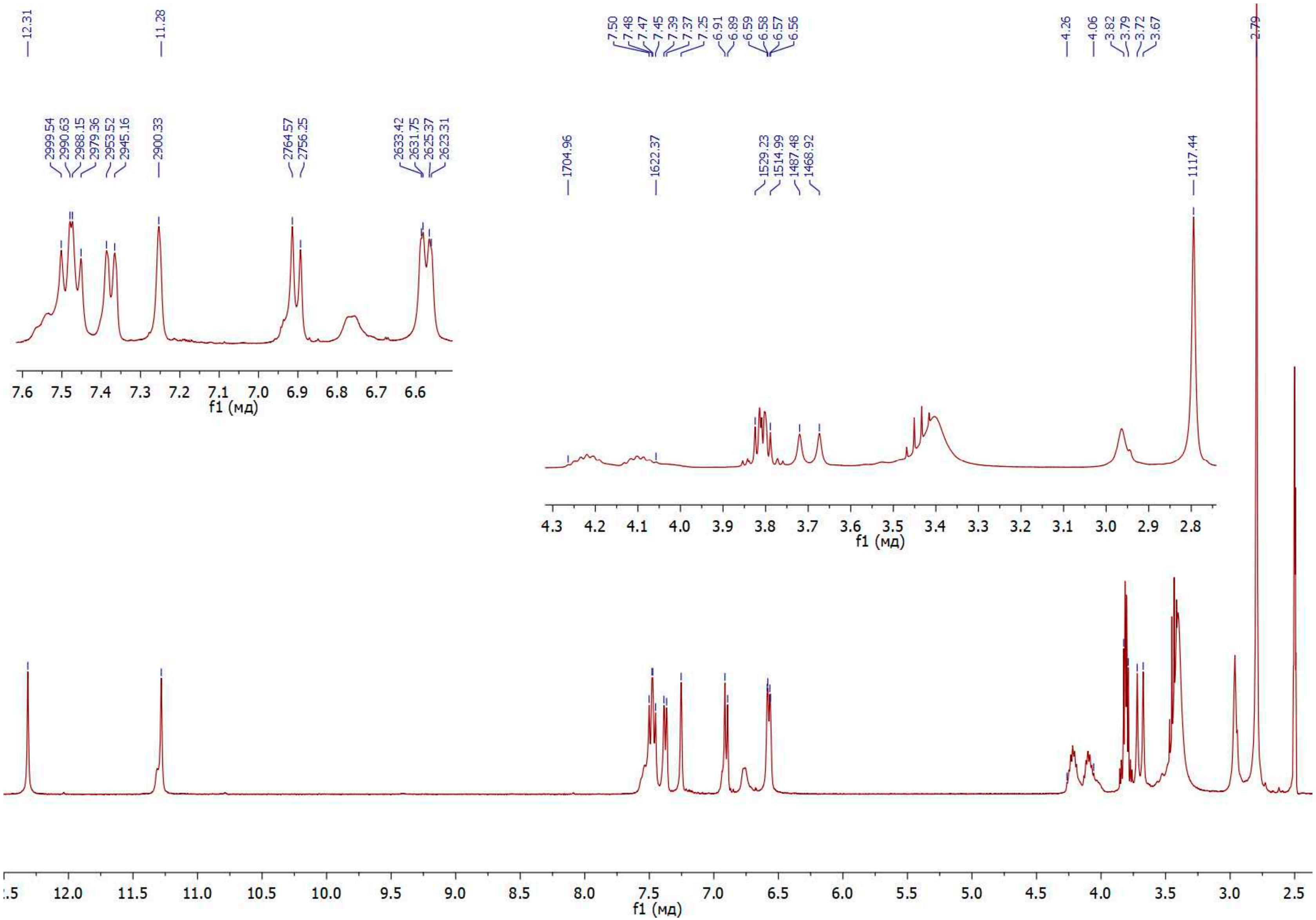

Figure S25. ^{1}H NMR spectrum of compound **5d** (400 MHz, DMSO-d_6)

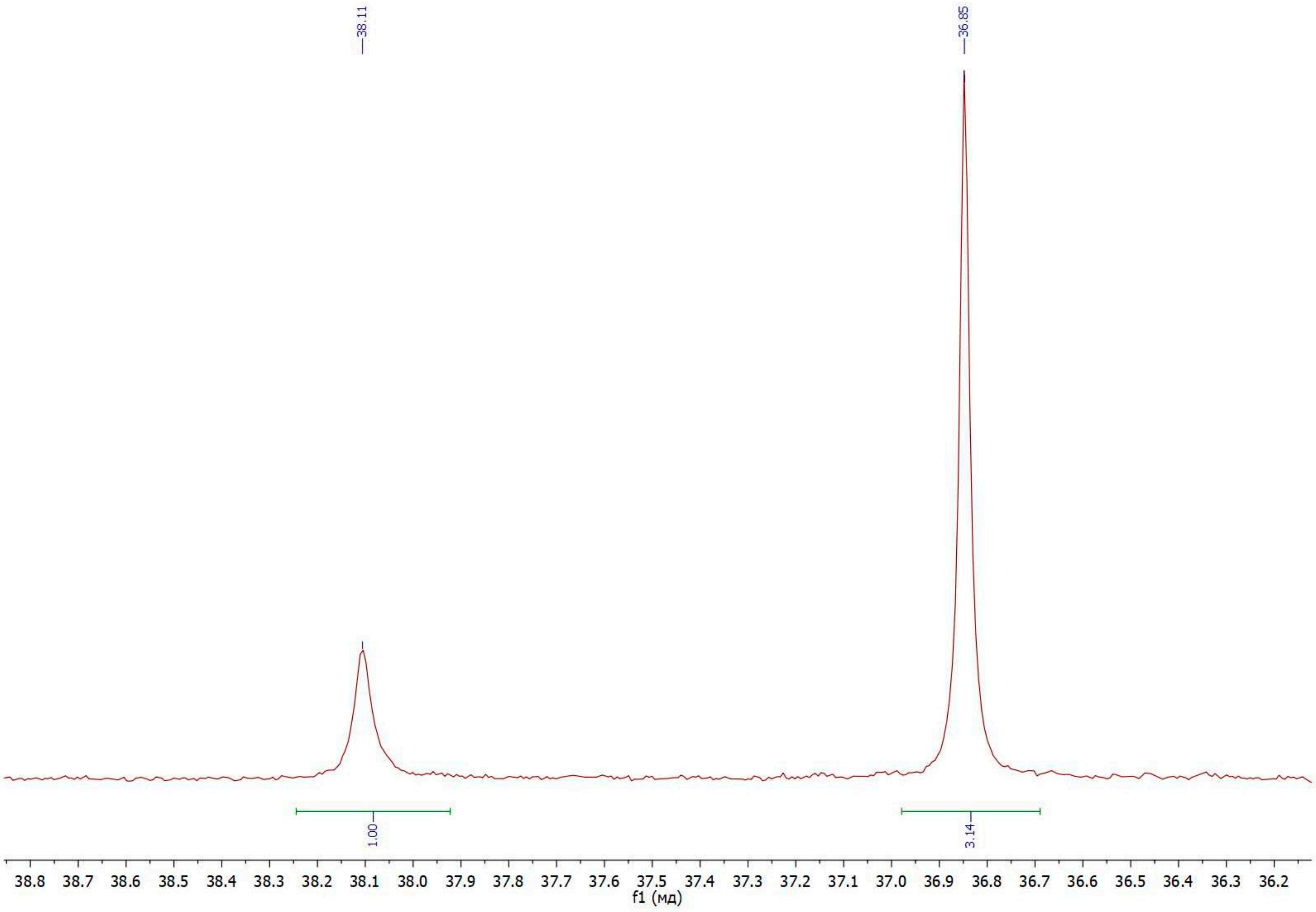

Figure S26. ^{31}P NMR spectrum of compound **5d** (243 MHz, DMSO-d_6)

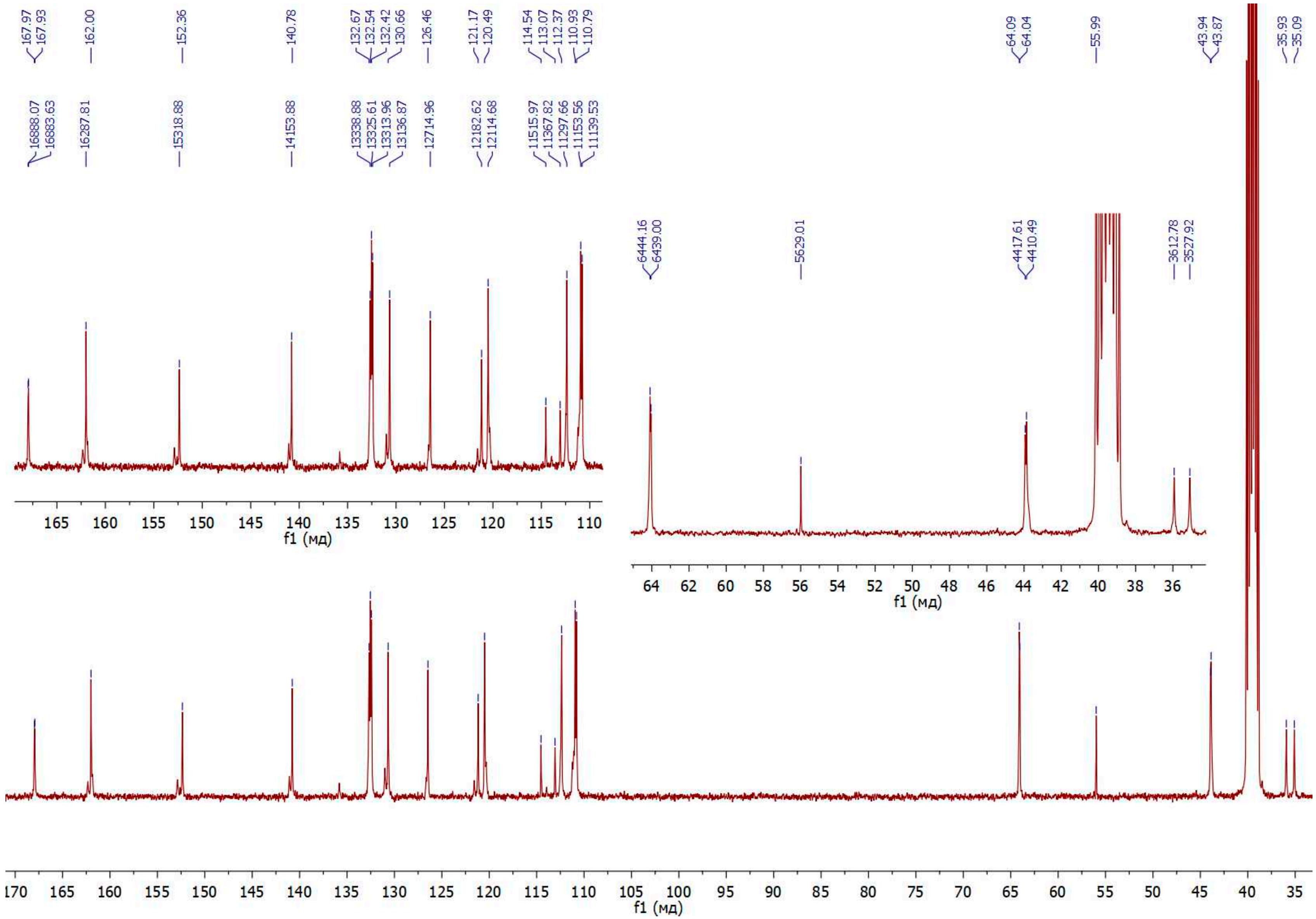

Figure S27. ^{13}C-{^{1}H} NMR spectrum of compound **5d** (101 MHz, DMSO-d_6)

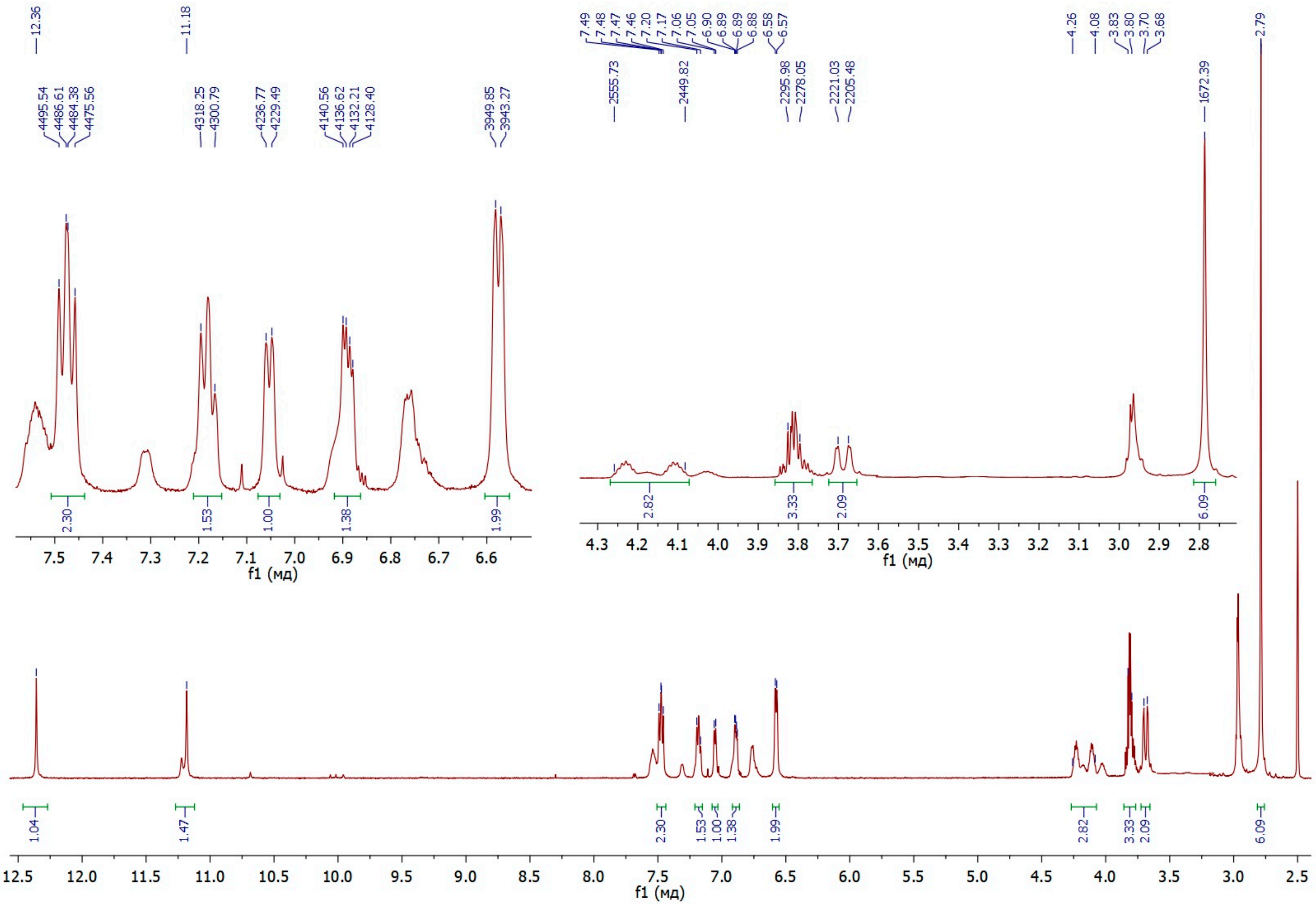

Figure S28. ^{1}H NMR spectrum of compound **5e** (400 MHz, DMSO-d_6)

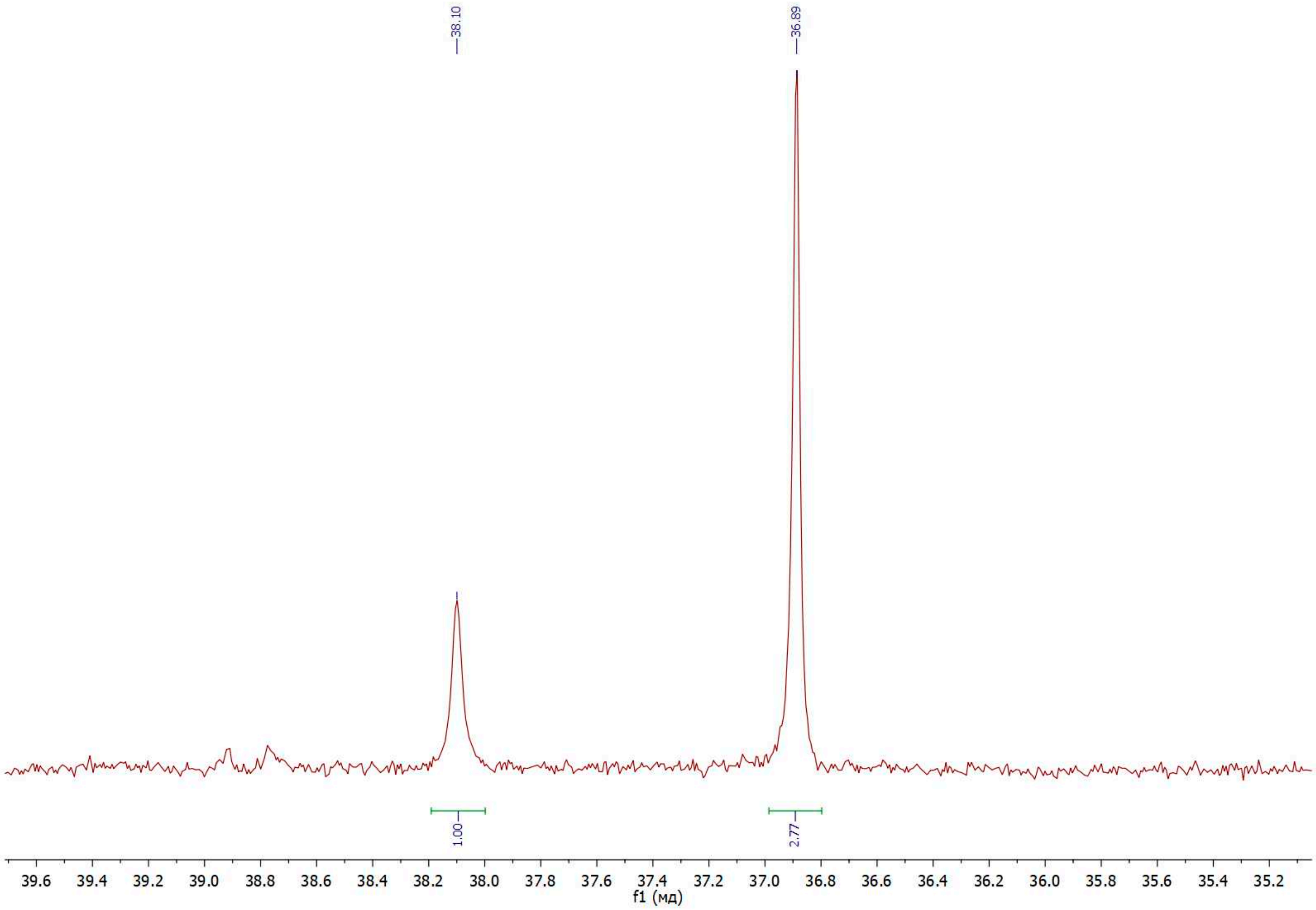

Figure S29. ^{31}P NMR spectrum of compound **5e** (243 MHz, DMSO-d_6)

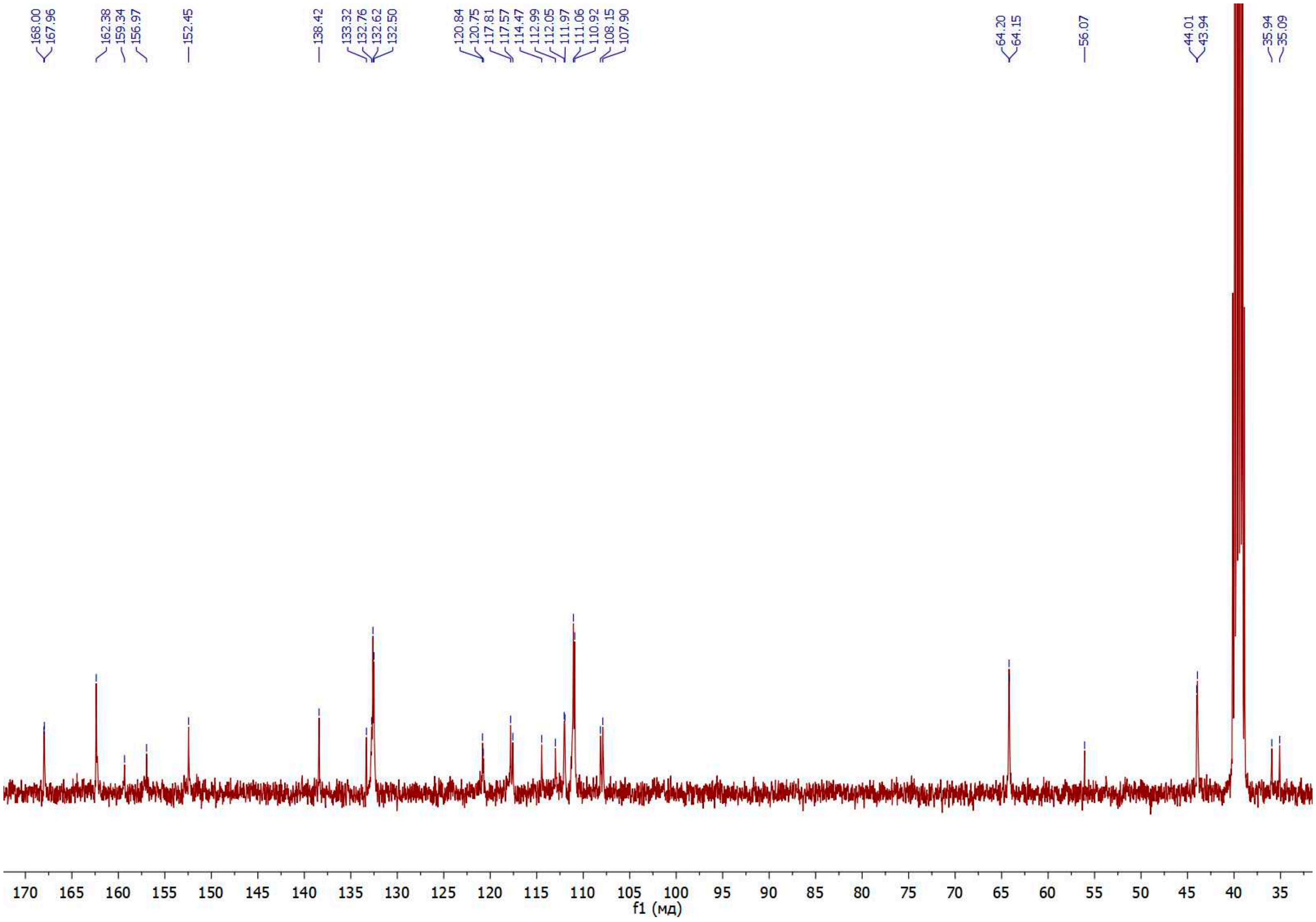

Figure S30. ^{13}C-{^{1}H} NMR spectrum of compound **5e** (101 MHz, DMSO-d_6)

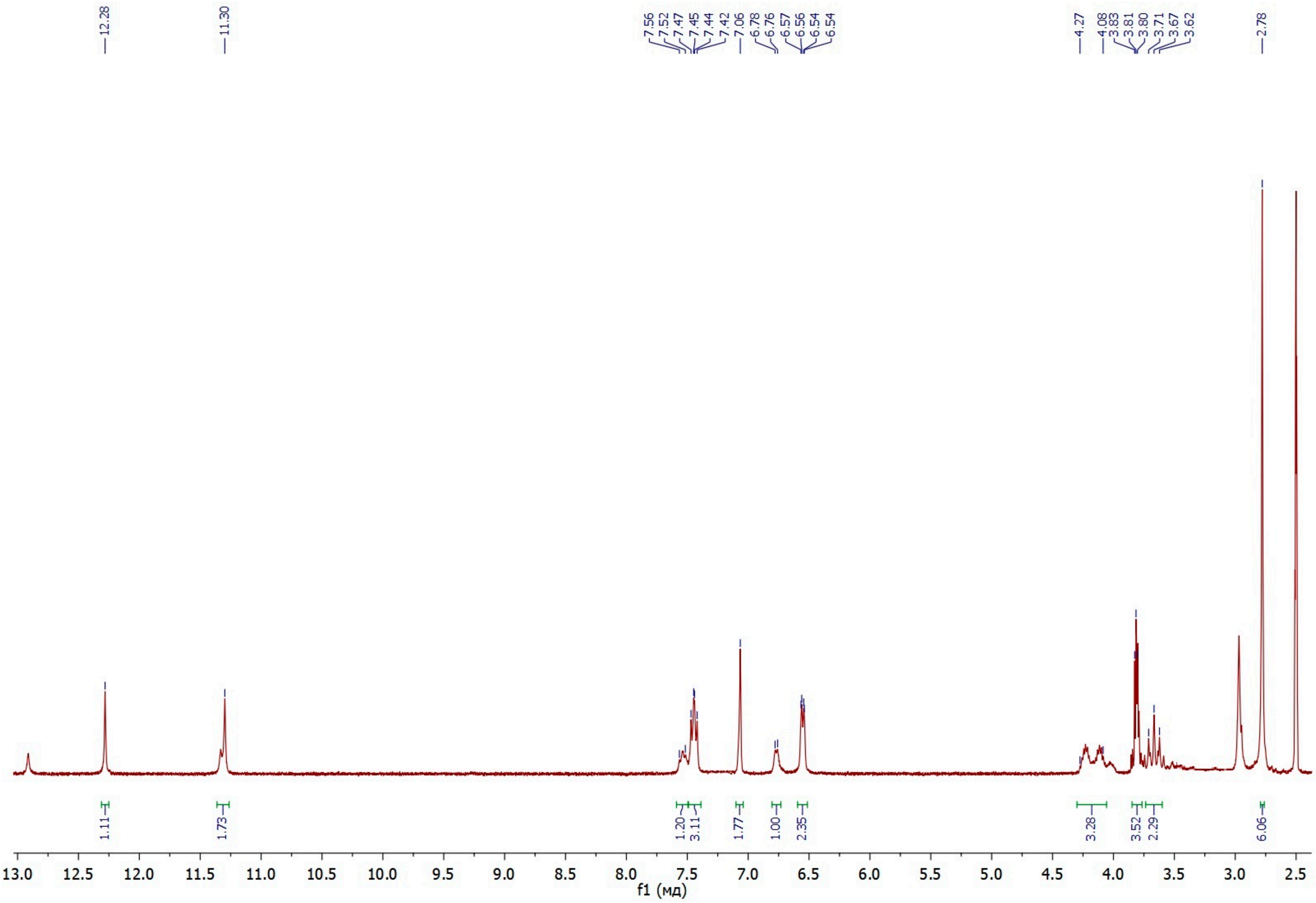

Figure S31. ^{1}H NMR spectrum of compound **5f** (400 MHz, DMSO-d_6)

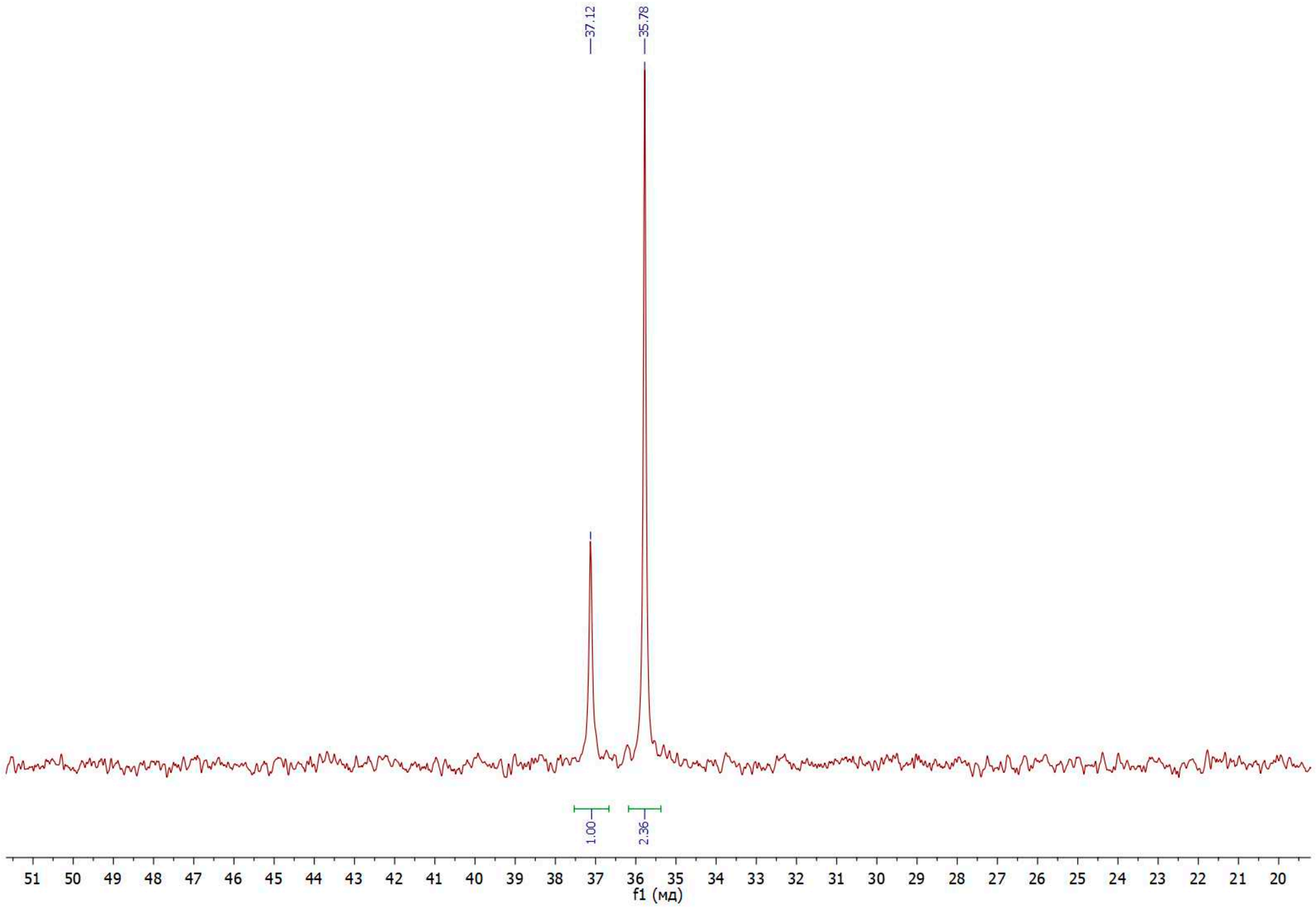

Figure S32. ^{31}P NMR spectrum of compound **5f** (162 MHz, DMSO-d_6)

6/23/2022 12:14:10

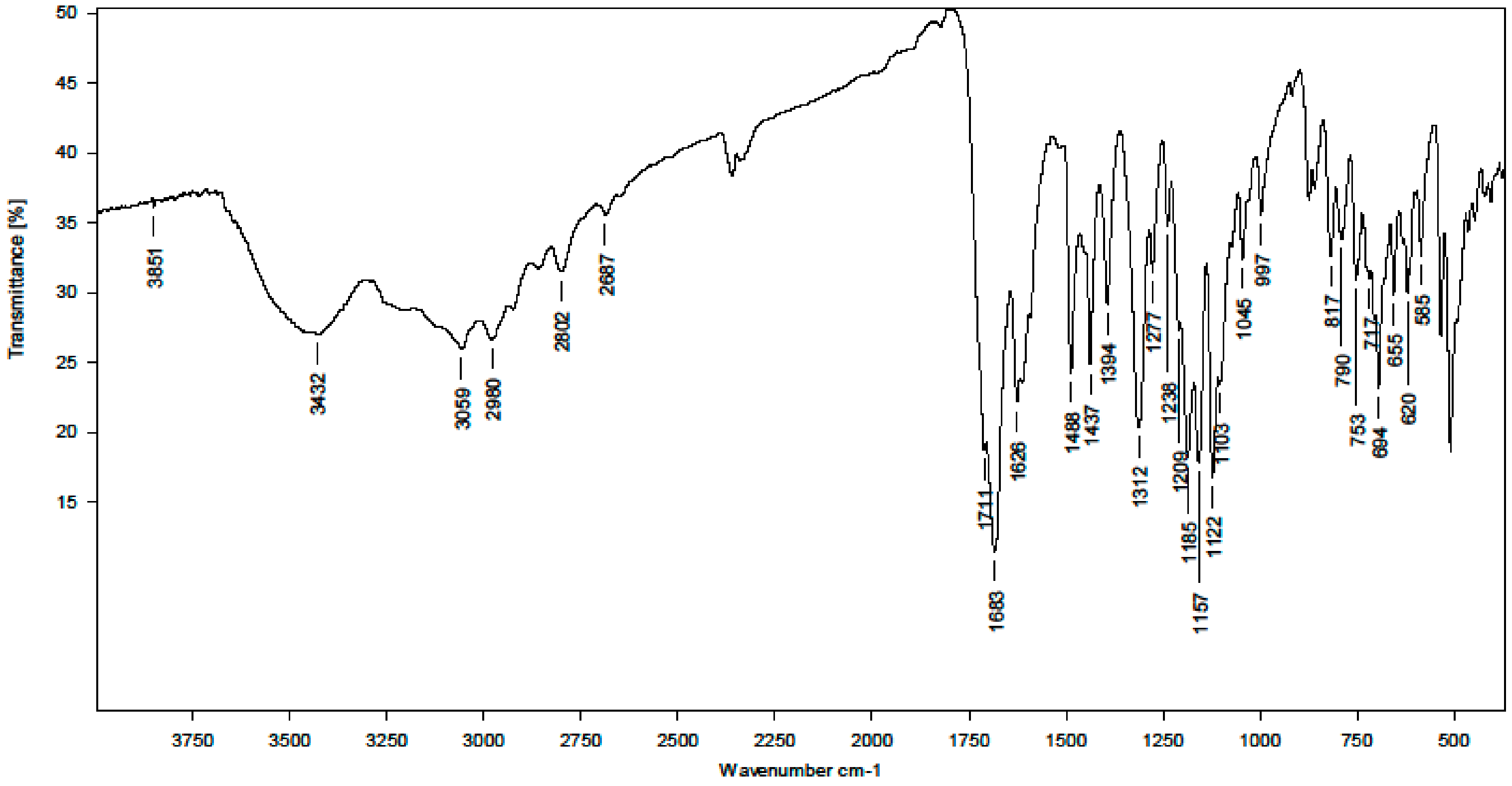

Sample Name C-11KAR

Operator Name Sasha

Instrument Type Tensor 27

Resolution 4

Path of File E:\work\2022

Date of Measurement 23/06/2022

Sample Form

Time of Measurement 1:12:36 PM

Filename C-11KAR.0

Figure S33. IR spectrum of compound **3a** (in KBr pellet)

11/16/2022 15:20:17

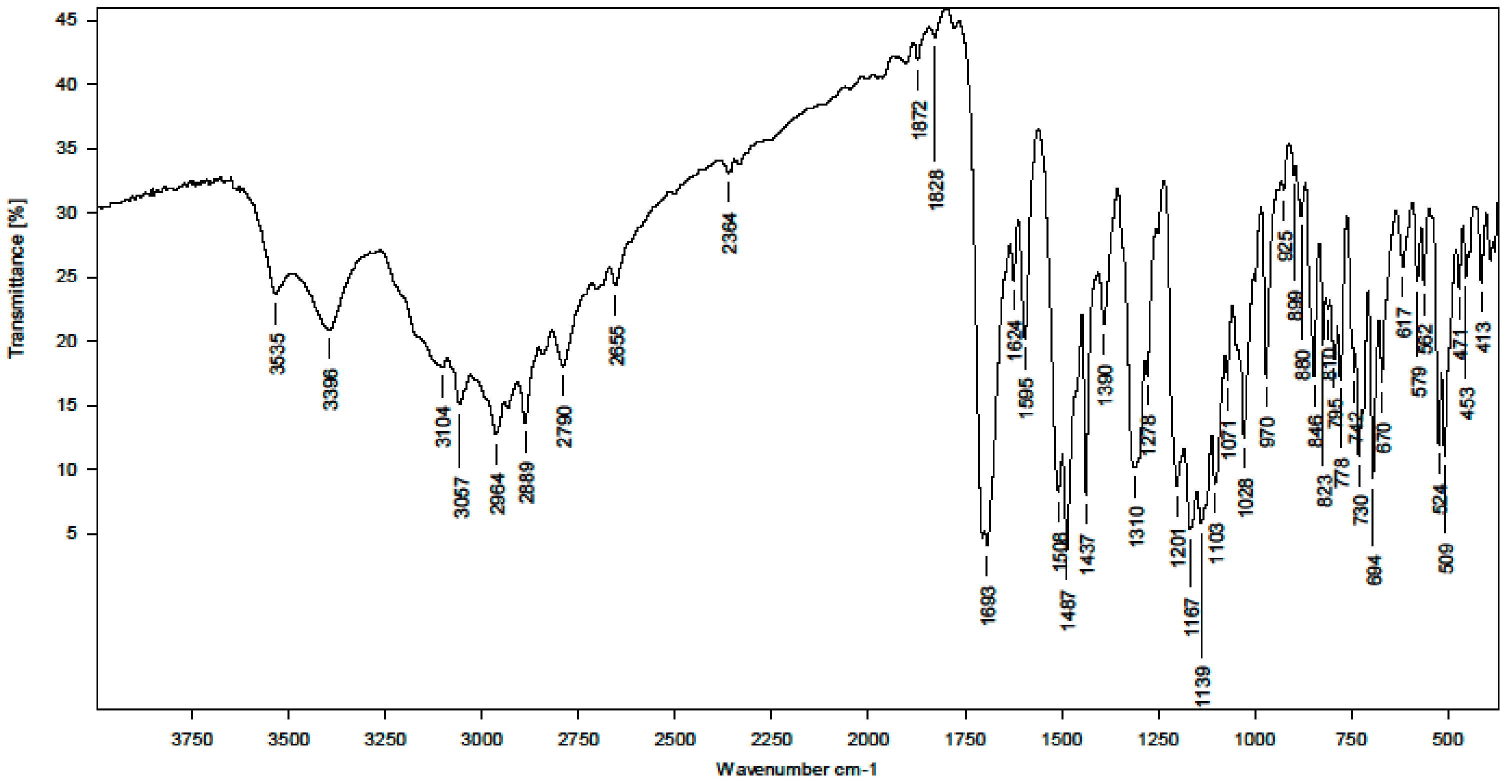

Sample Name C-15Kap

Path of File E:\work\2022

Filename C-15Kap.0

Operator Name Sasha

Date of Measurement 16/11/2022

Instrument Type Tensor 27

Sample Form

Resolution 4

Time of Measurement 3:19:14 PM

Figure S34. IR spectrum of compound **3b** (in KBr pellet)

11/16/2022 13:10:45

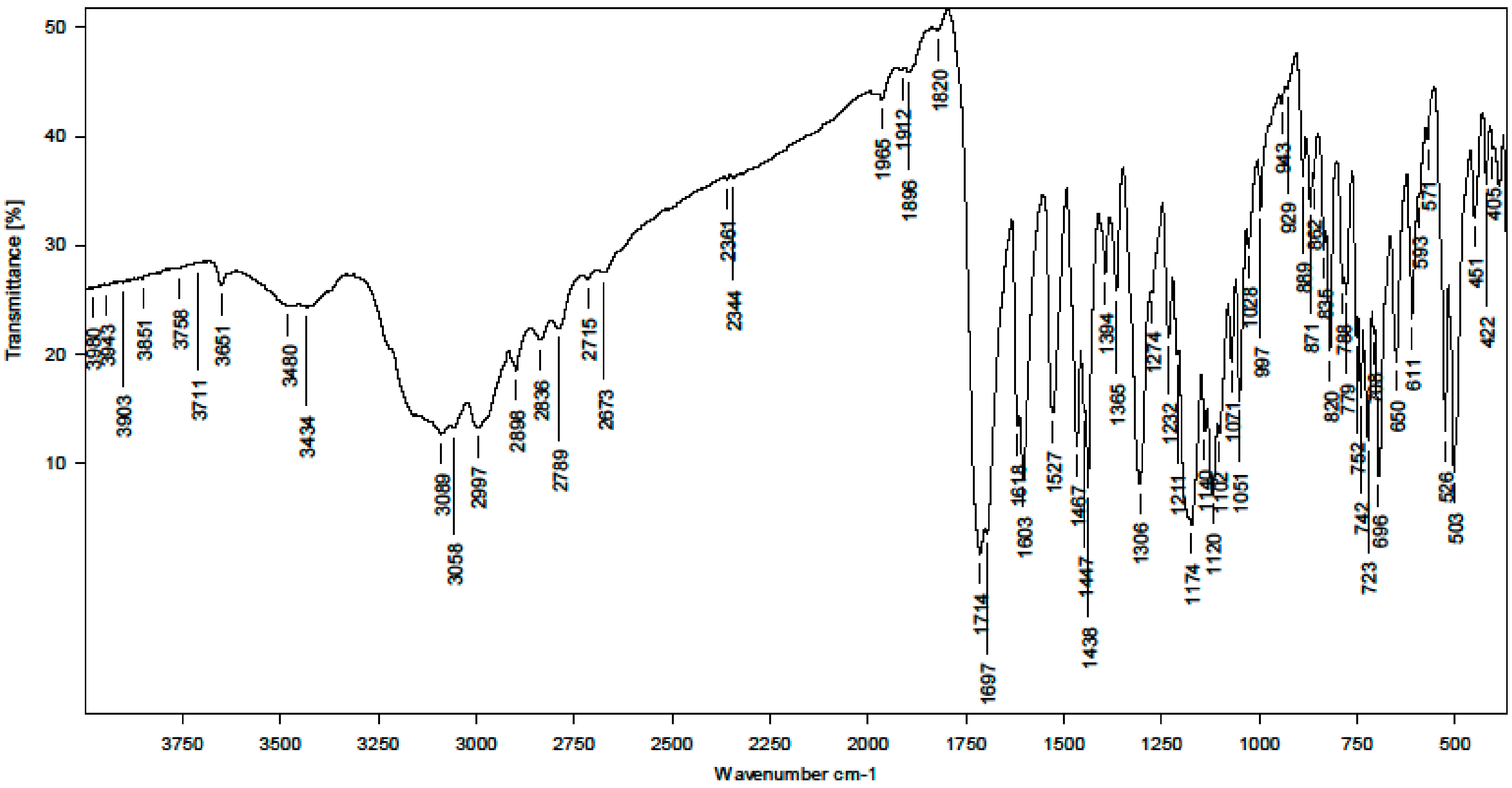

Sample Name C-14Kap

Path of File E:\work\2022

Filename C-14Kap.0

Operator Name Sasha

Date of Measurement 16/11/2022

Instrument Type Tensor 27

Sample Form

Resolution 4

Time of Measurement 1:09:43 PM

Figure S35. IR spectrum of compound **3c** (in KBr pellet)

6/23/2022 12:04:37

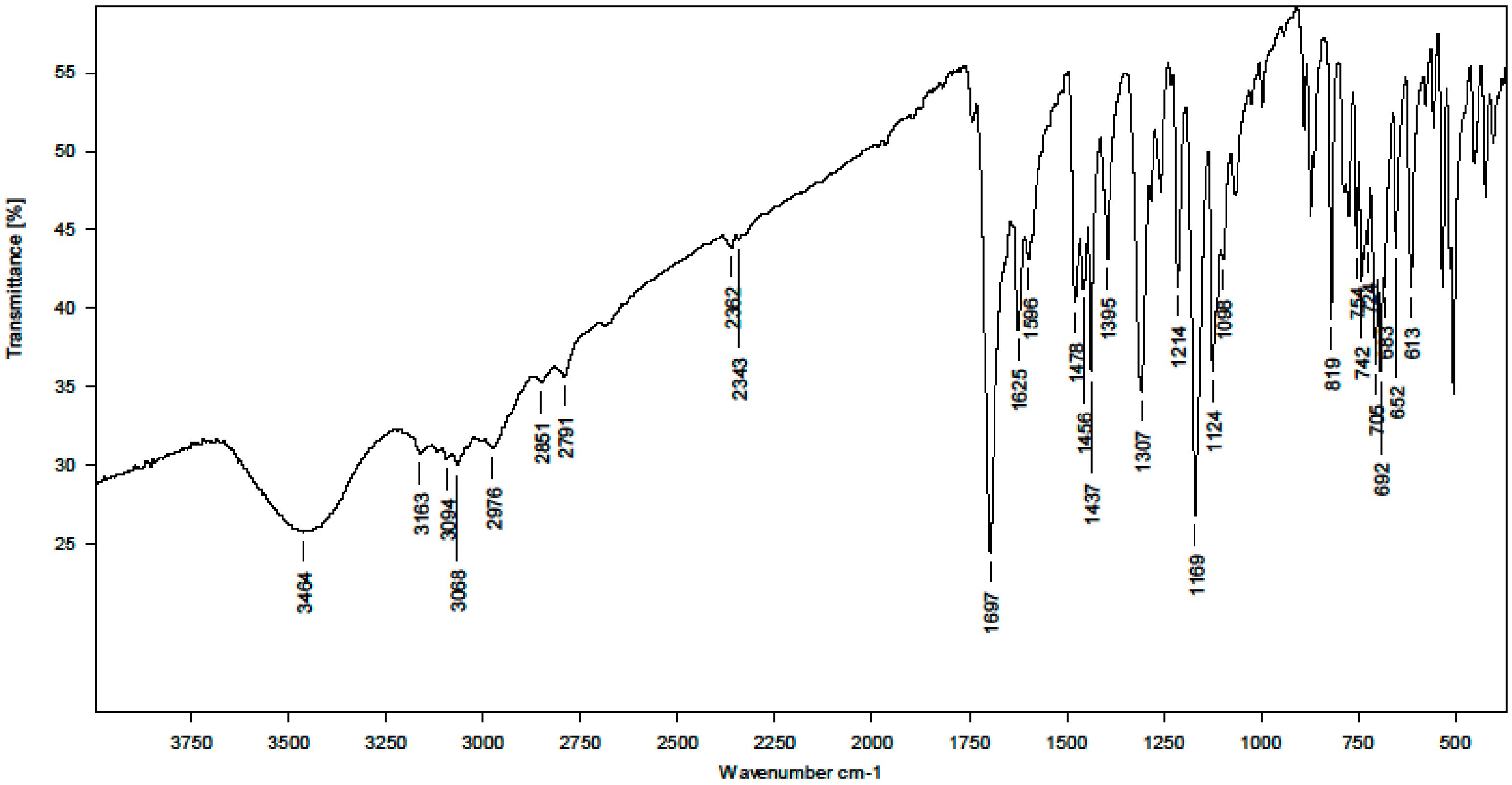

Sample Name C-10KAR

Operator Name Sasha

Instrument Type Tensor 27

Resolution 4

Path of File E:\work\2022

Date of Measurement 23/06/2022

Sample Form

Time of Measurement 1:03:39 PM

Filename C-10KAR.0

Figure S36. IR spectrum of compound **3d** (in KBr pellet)

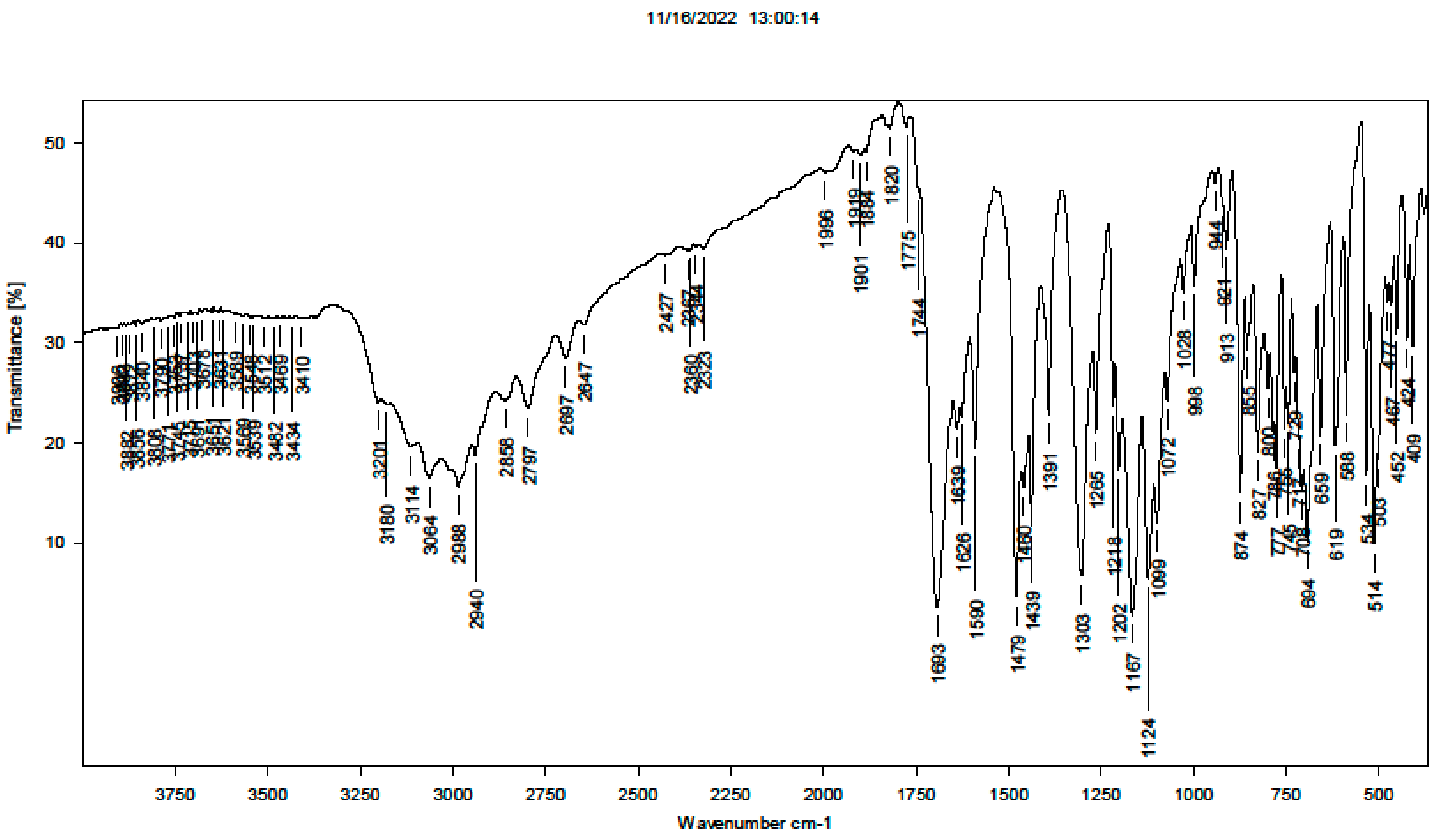

Sample Name C-13Kap

Path of File E:\work\2022

Filename C-13Kap.0

Operator Name Sasha

Date of Measurement 16/11/2022

Instrument Type Tensor 27

Sample Form

Resolution 4

Time of Measurement 12:56:44 PM

Figure S37. IR spectrum of compound **3e** (in KBr pellet)

6/9/2022 13:12:56

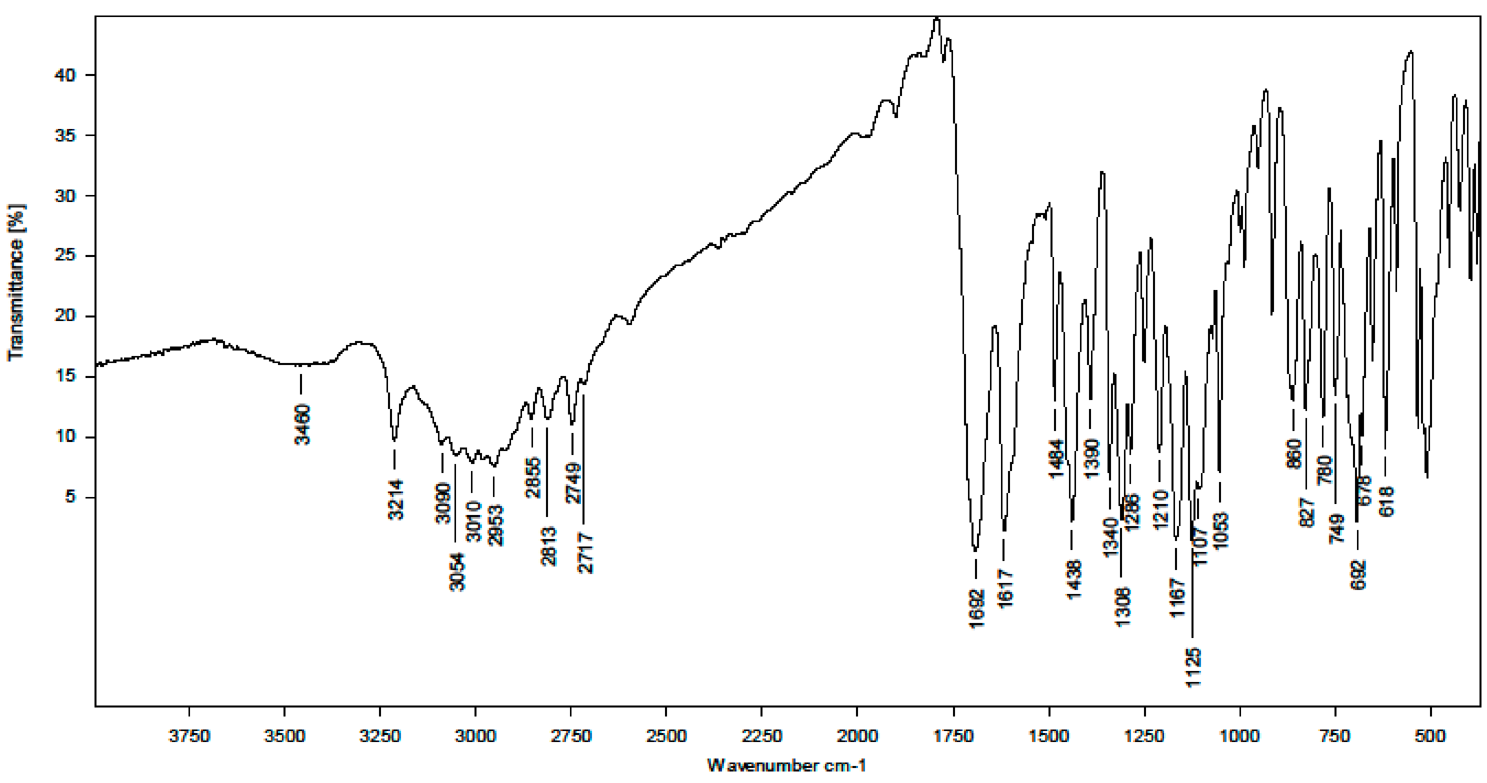

Sample Name C-8_1

Operator Name Sasha

Instrument Type Tensor 27

Resolution 4

Path of File E:\work\2022

Date of Measurement 09/06/2022

Sample Form

Time of Measurement 2:09:14 PM

Filename C-8_1.0

Figure S38. IR spectrum of compound **3f** (in KBr pellet)

01.02.2022 15:31:17

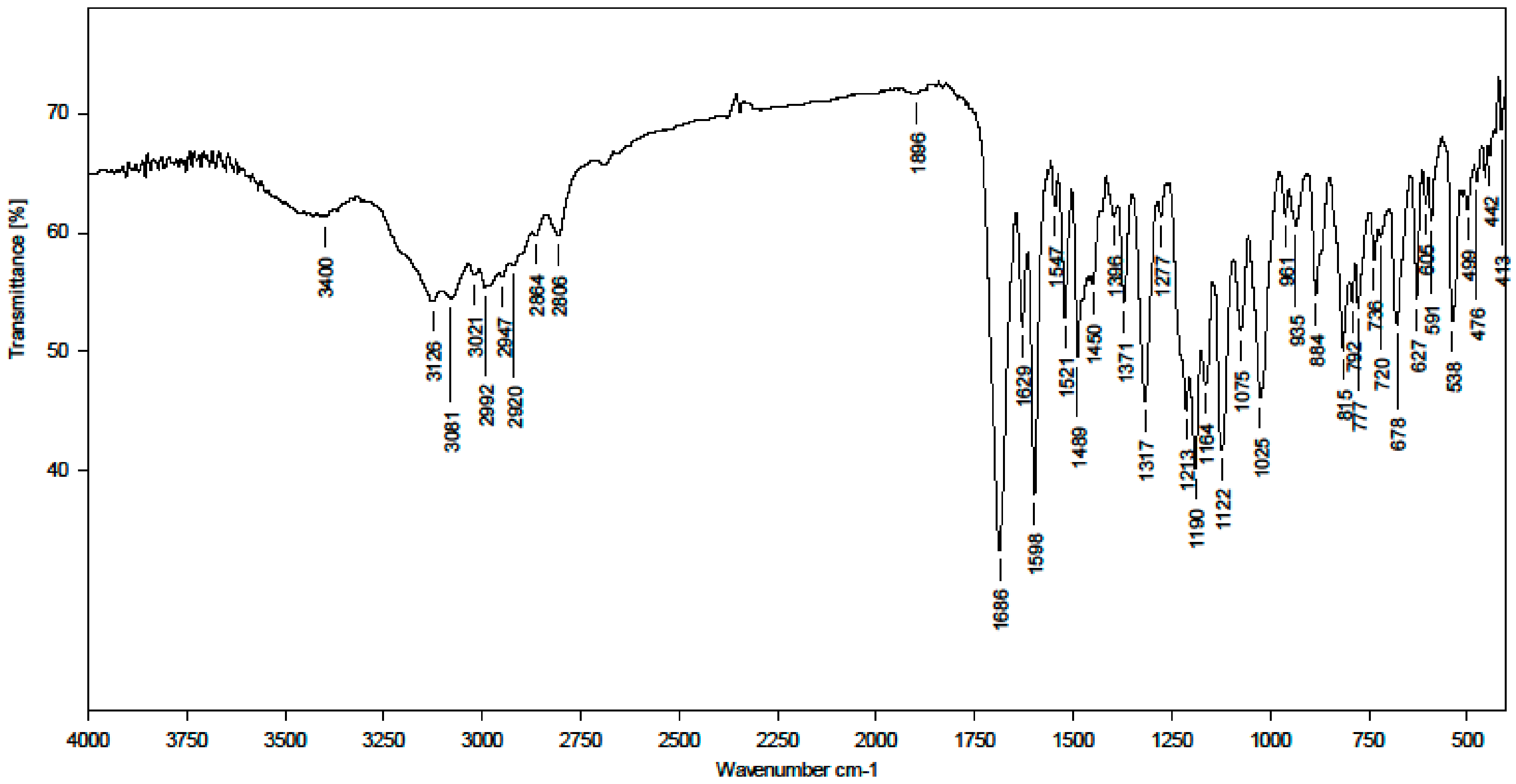

Sample Name C4Kar

Operator Name Default

Instrument Type VECTOR22

Resolution 4

Path of File E:\Work\2022

Date of Measurement 01.02.2022

Sample Form

Time of Measurement 15:29:14 (GMT+3)

Filename C4Kar.0

Figure S39. IR spectrum of compound **5a** (in KBr pellet)

24.01.2022 14:54:04

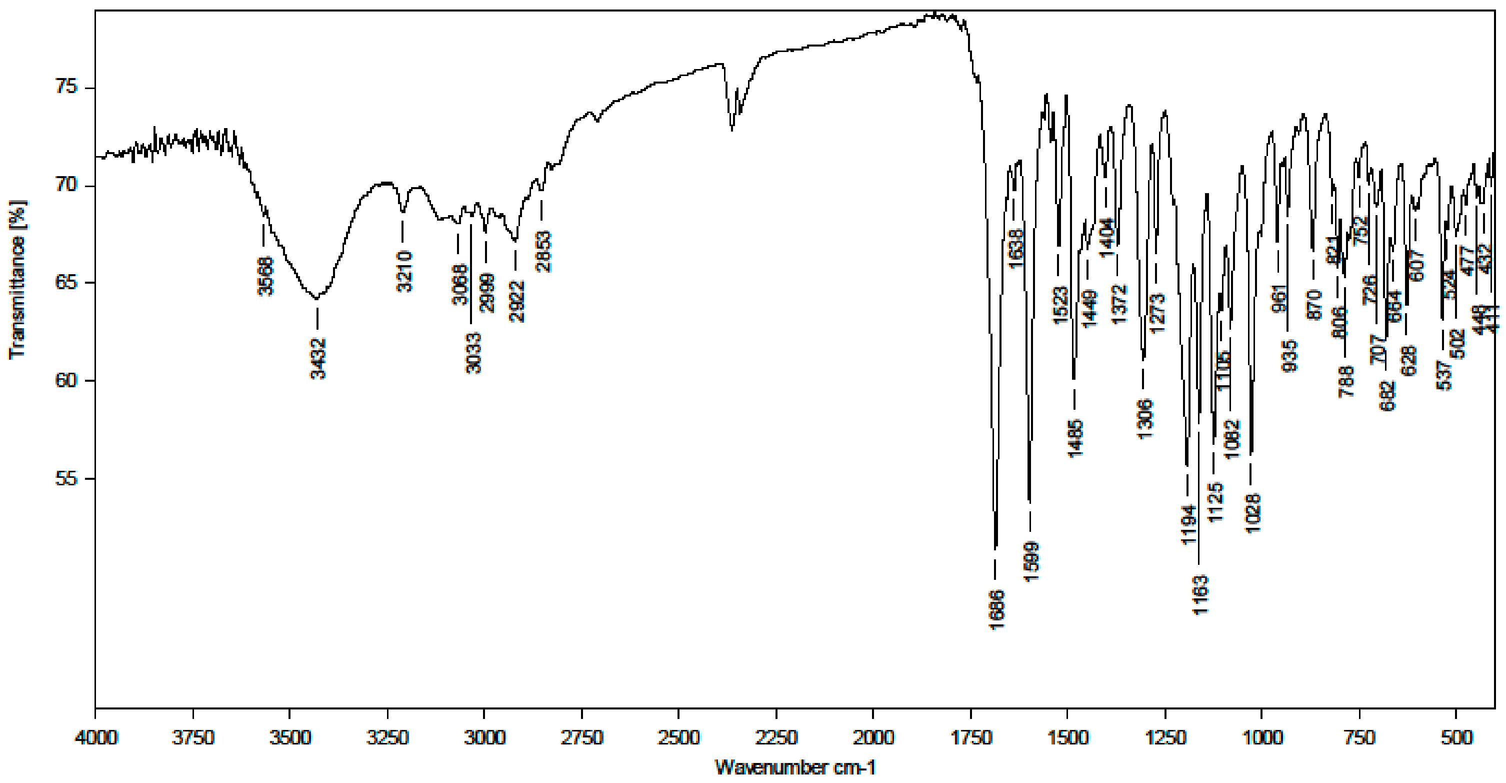

Sample Name Ñ-2Kar

Path of File E:\Work\2022

Filename Ñ-2Kar.0

Operator Name Default

Date of Measurement 24.01.2022

Instrument Type VECTOR22

Sample Form

Resolution 4

Time of Measurement 14:51:13 (GMT+3)

Figure S40. IR spectrum of compound **5b** (in KBr pellet)

22.04.2022 14:25:23

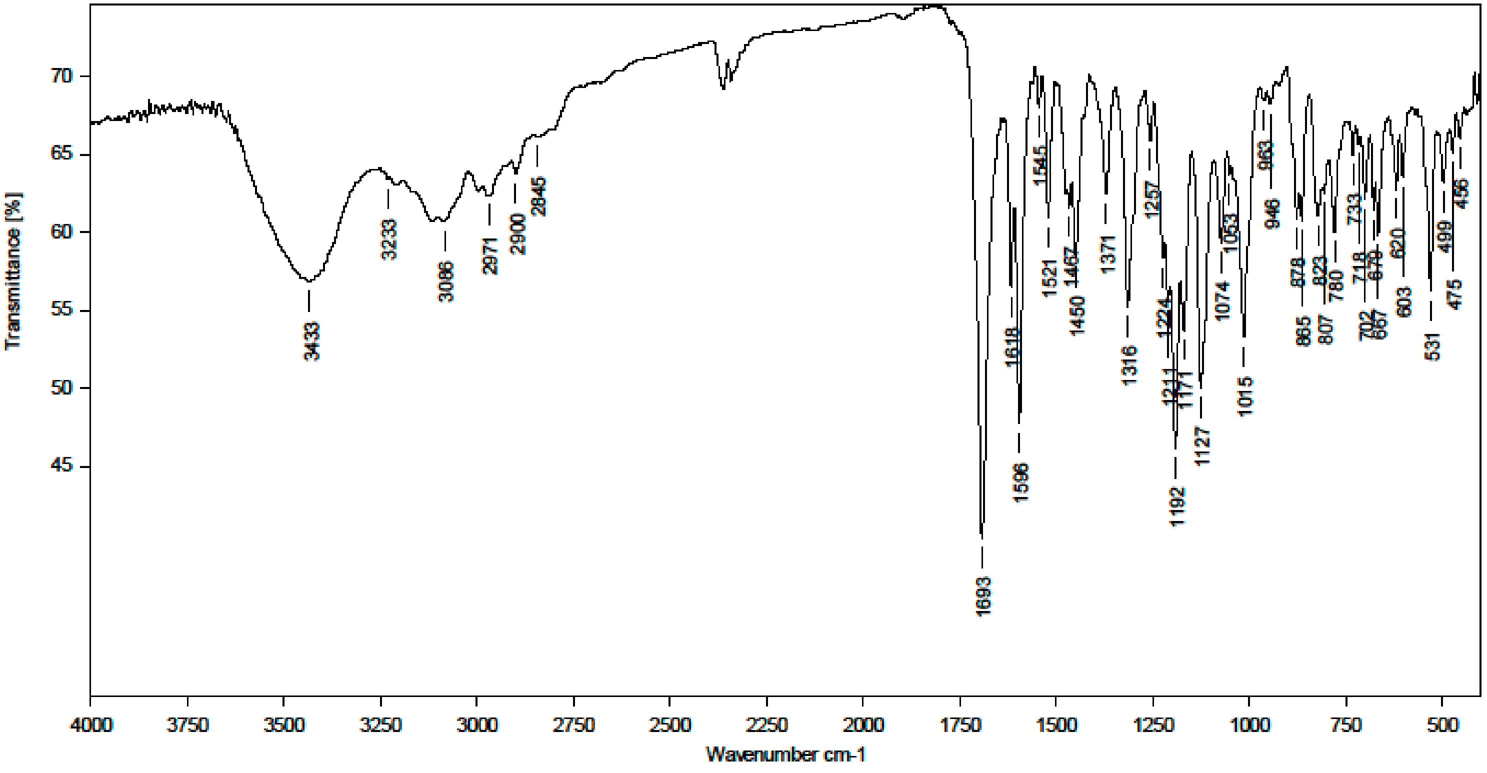

Sample Name C-6kar

Operator Name Default

Instrument Type VECTOR22

Resolution 4

Path of File E:\Work\2022

Date of Measurement 22.04.2022

Sample Form

Time of Measurement 14:23:08 (GMT+3)

Filename C-6kar.0

Figure S41. IR spectrum of compound **5c** (in KBr pellet)

31.05.2022 15:56:28

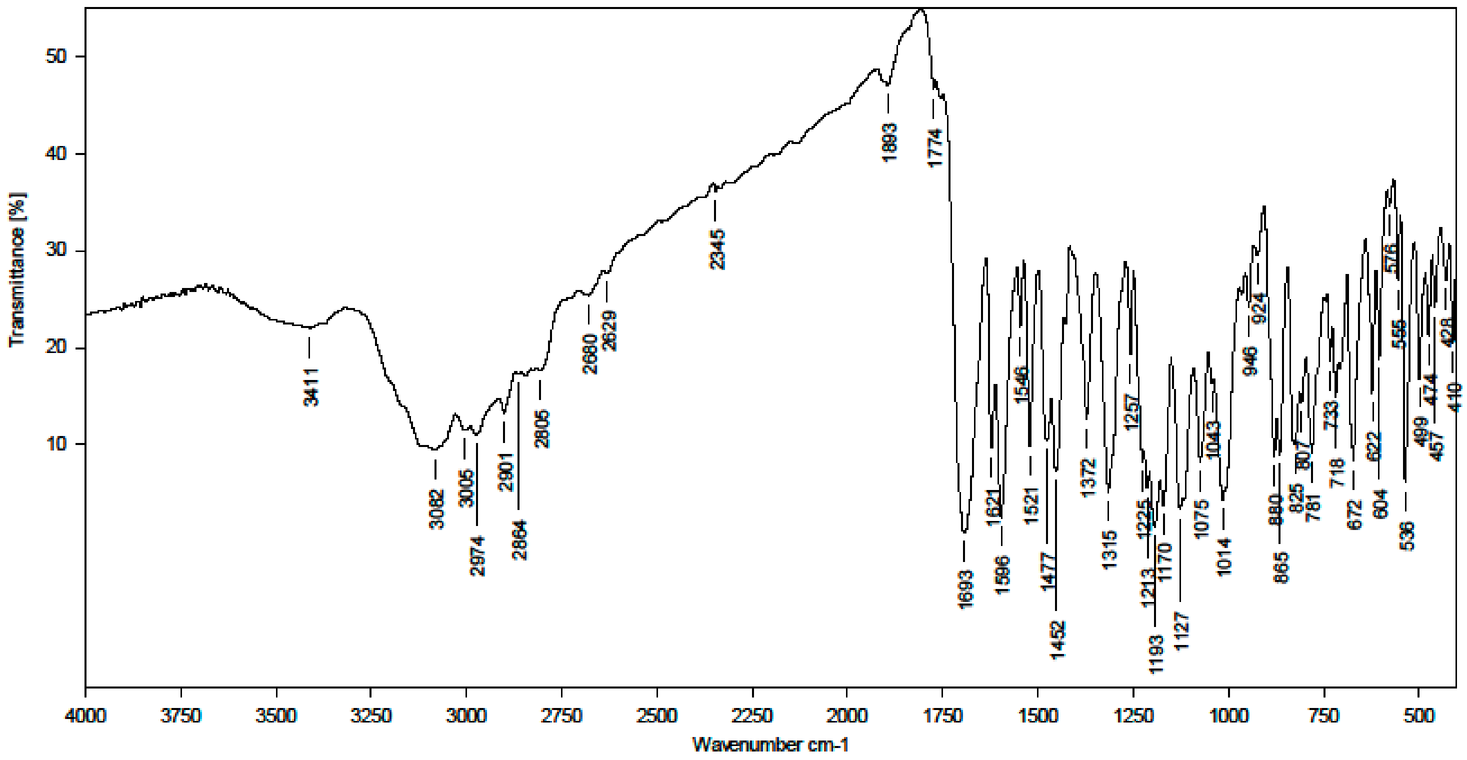

Sample Name C-5Kar

Operator Name Default

Instrument Type VECTOR22

Resolution 4

Path of File E:\Work\2022

Date of Measurement 31.05.2022

Sample Form

Time of Measurement 15:55:01 (GMT+3)

Filename C-5Kar.0

Figure S42. IR spectrum of compound **5d** (in KBr pellet)

31.05.2022 11:29:13

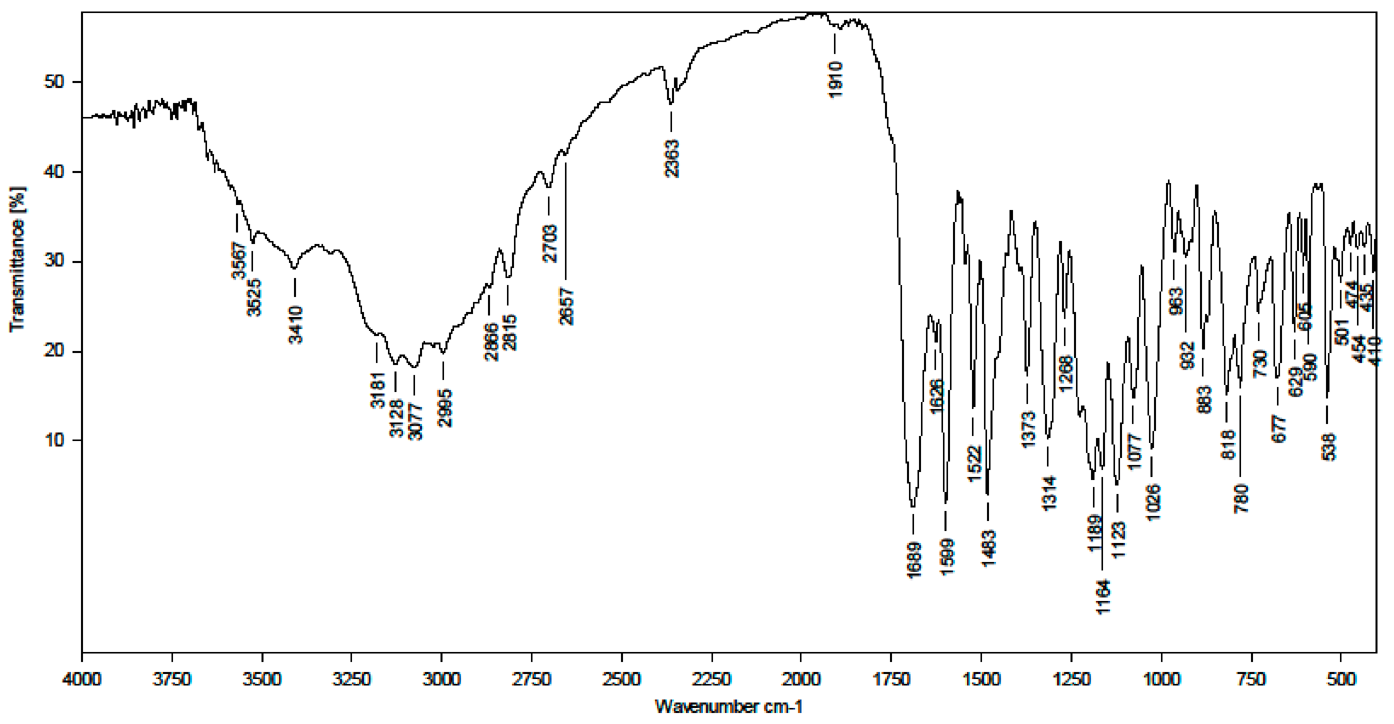

Sample Name Ñ-7Kar

Operator Name Default

Instrument Type VECTOR22

Resolution 4

Path of File E:\Work\2022

Date of Measurement 31.05.2022

Sample Form

Time of Measurement 11:18:33 (GMT+3)

Filename Ñ-7Kar.0

Figure S43. IR spectrum of compound **5e** (in KBr pellet)

01.02.2022 15:40:52

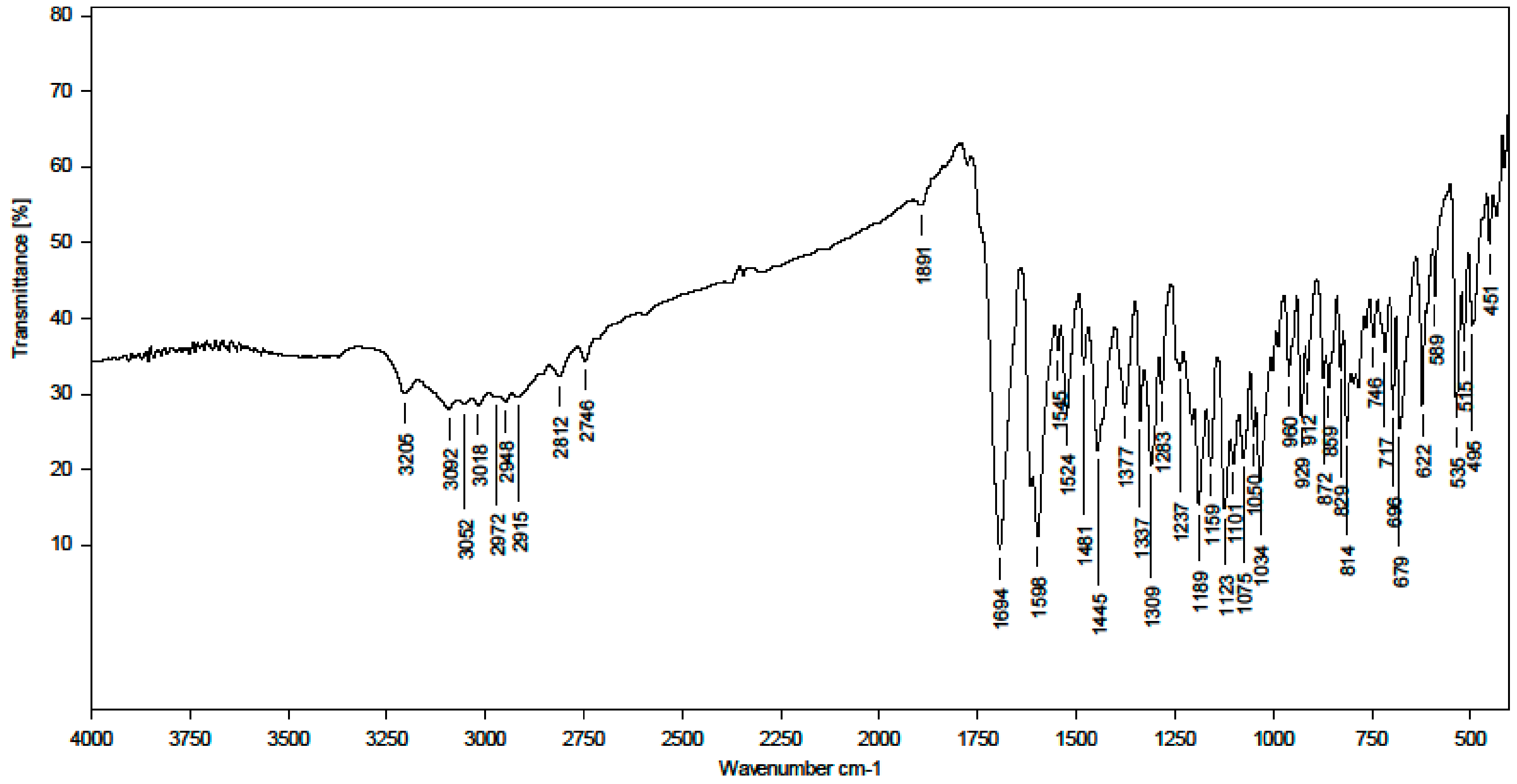

Sample Name C3Kar

Path of File E:\Work\2022

Filename C3Kar.0

Operator Name Default

Date of Measurement 01.02.2022

Instrument Type VECTOR22

Sample Form

Resolution 4

Time of Measurement 15:39:35 (GMT+3)

Figure S44. IR spectrum of compound **5f** (in KBr pellet)

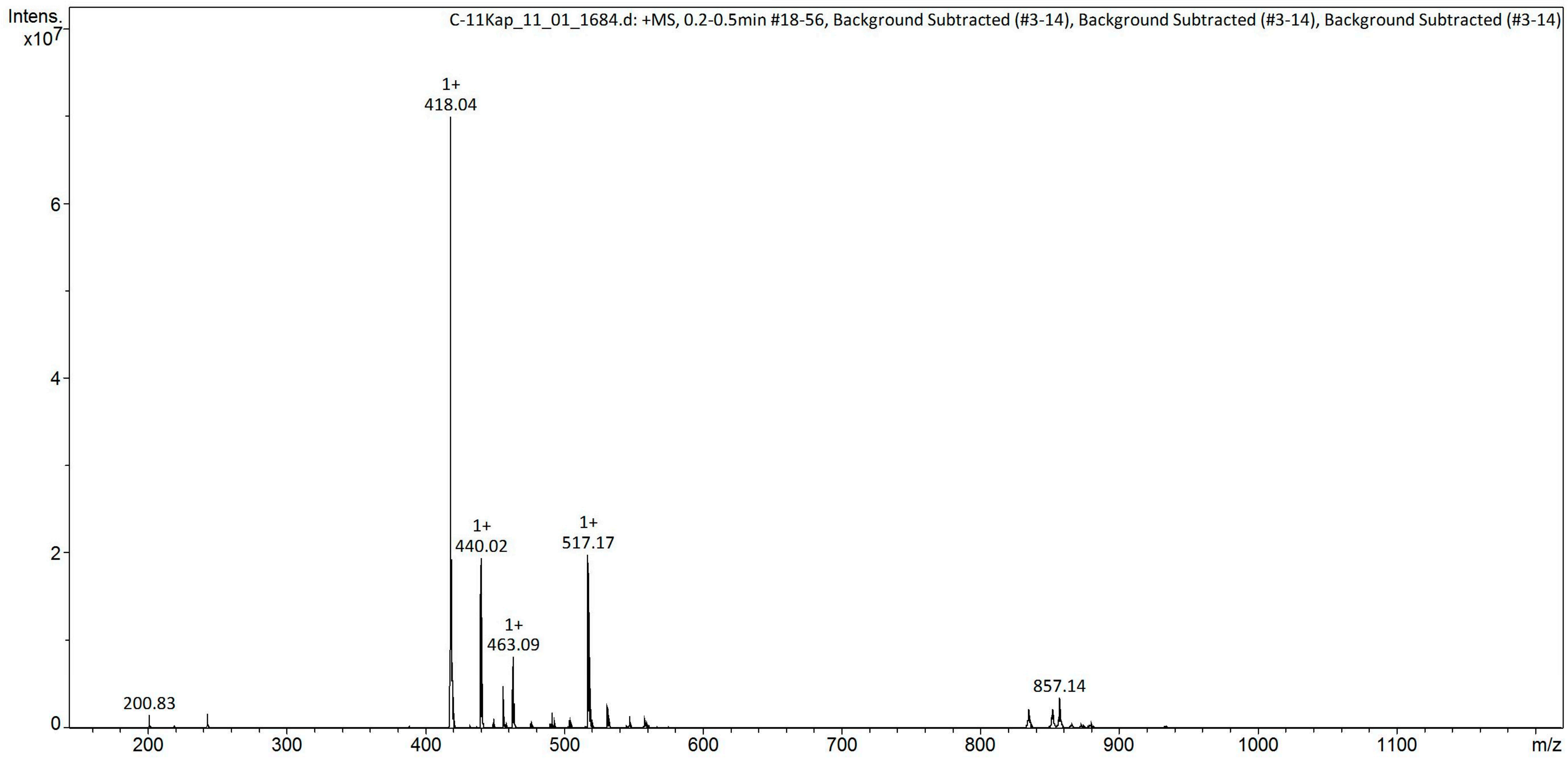

Bruker Compass DataAnalysis 4.4 printed: 7/4/2022 10:17:39 AM Page 1 of 1

Figure S45. ESI spectrum of compound **3a**

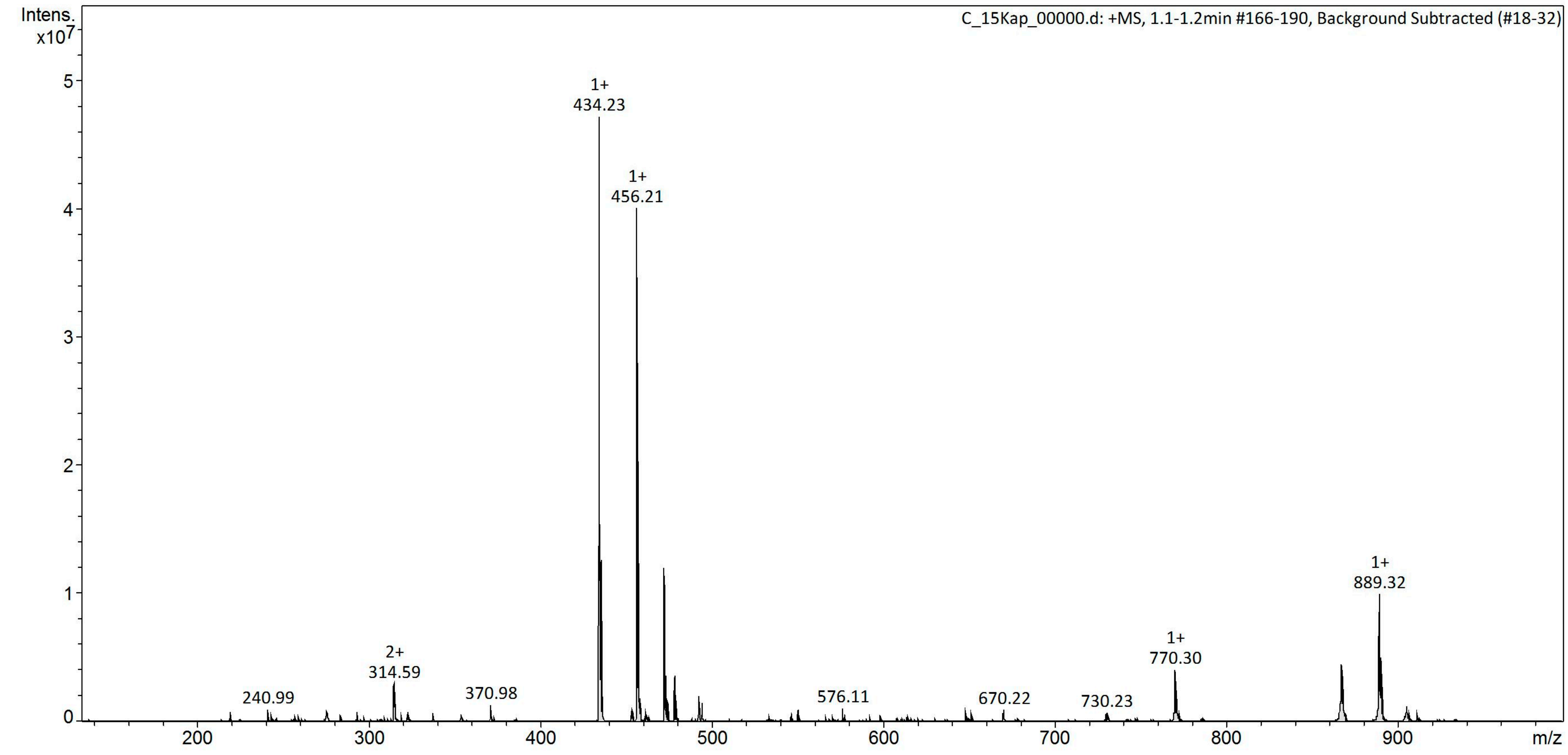

Bruker Compass DataAnalysis 4.4 printed: 1/26/2023 10:37:32 AM Page 1 of 1

Figure S46. ESI spectrum of compound **3b**

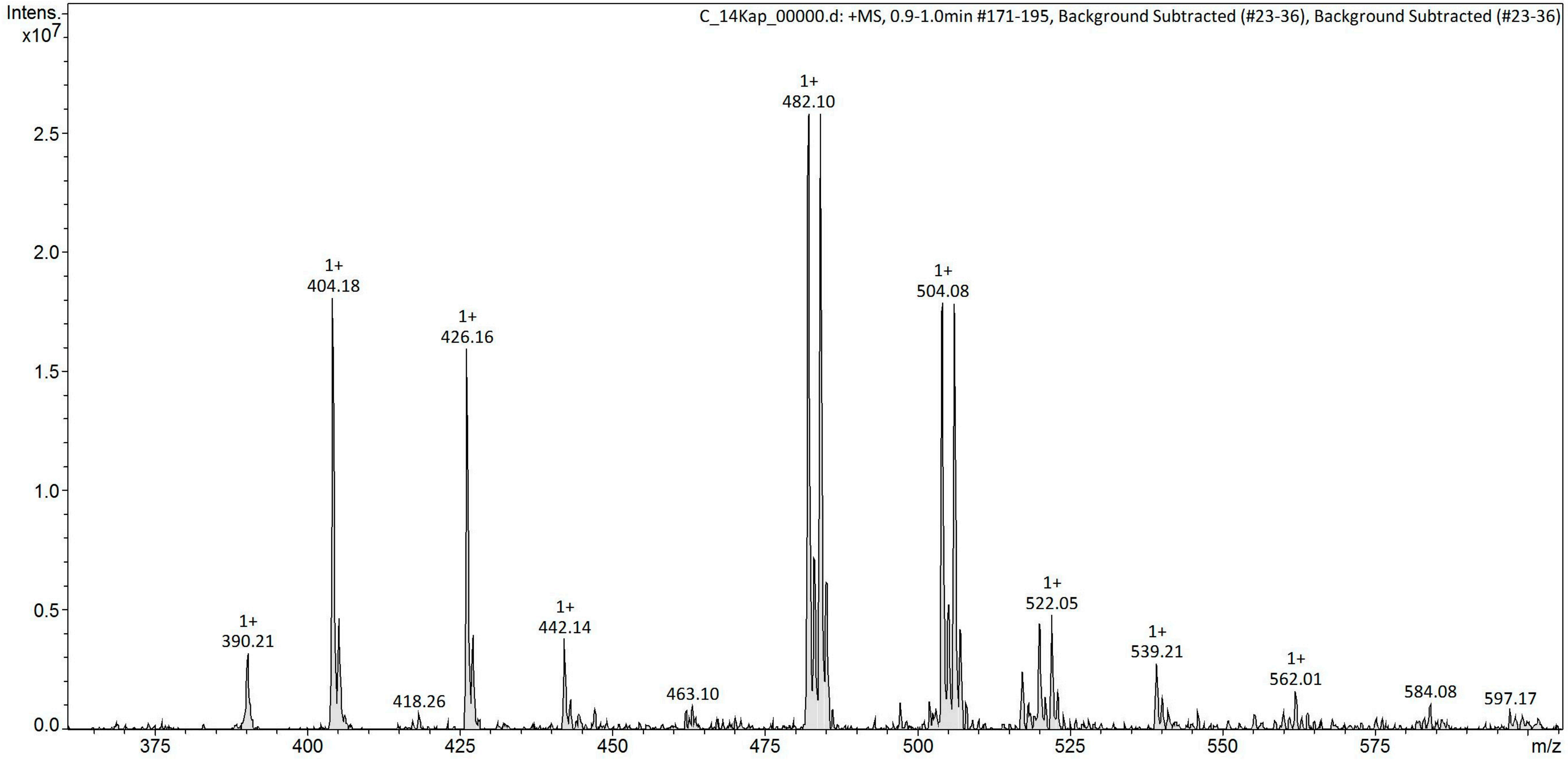

Bruker Compass DataAnalysis 4.4 printed: 10/25/2022 11:03:37 AM Page 1 of 1

Figure S47. ESI spectrum of compound **3c**

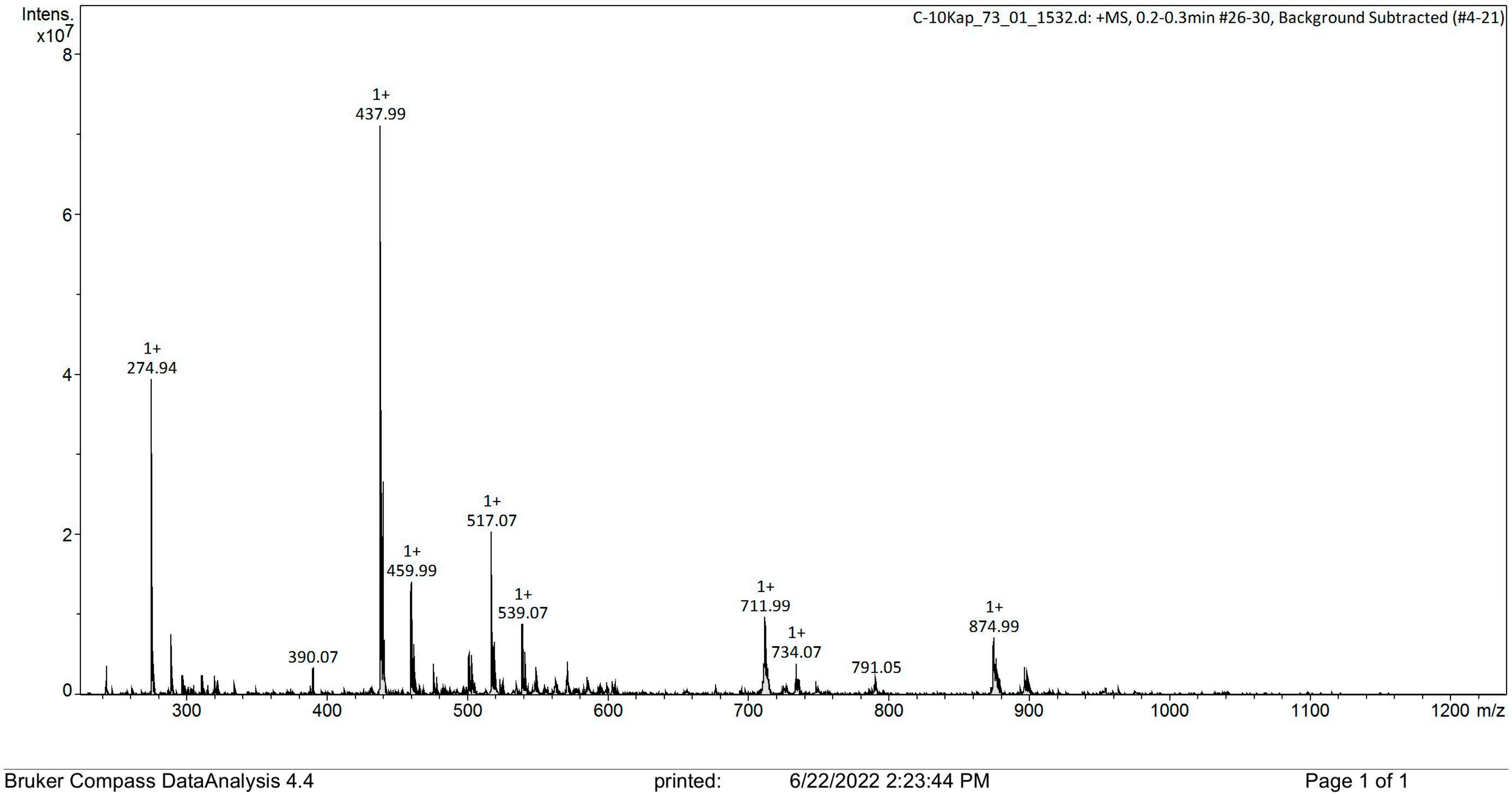

Figure S48. ESI spectrum of compound **3d**

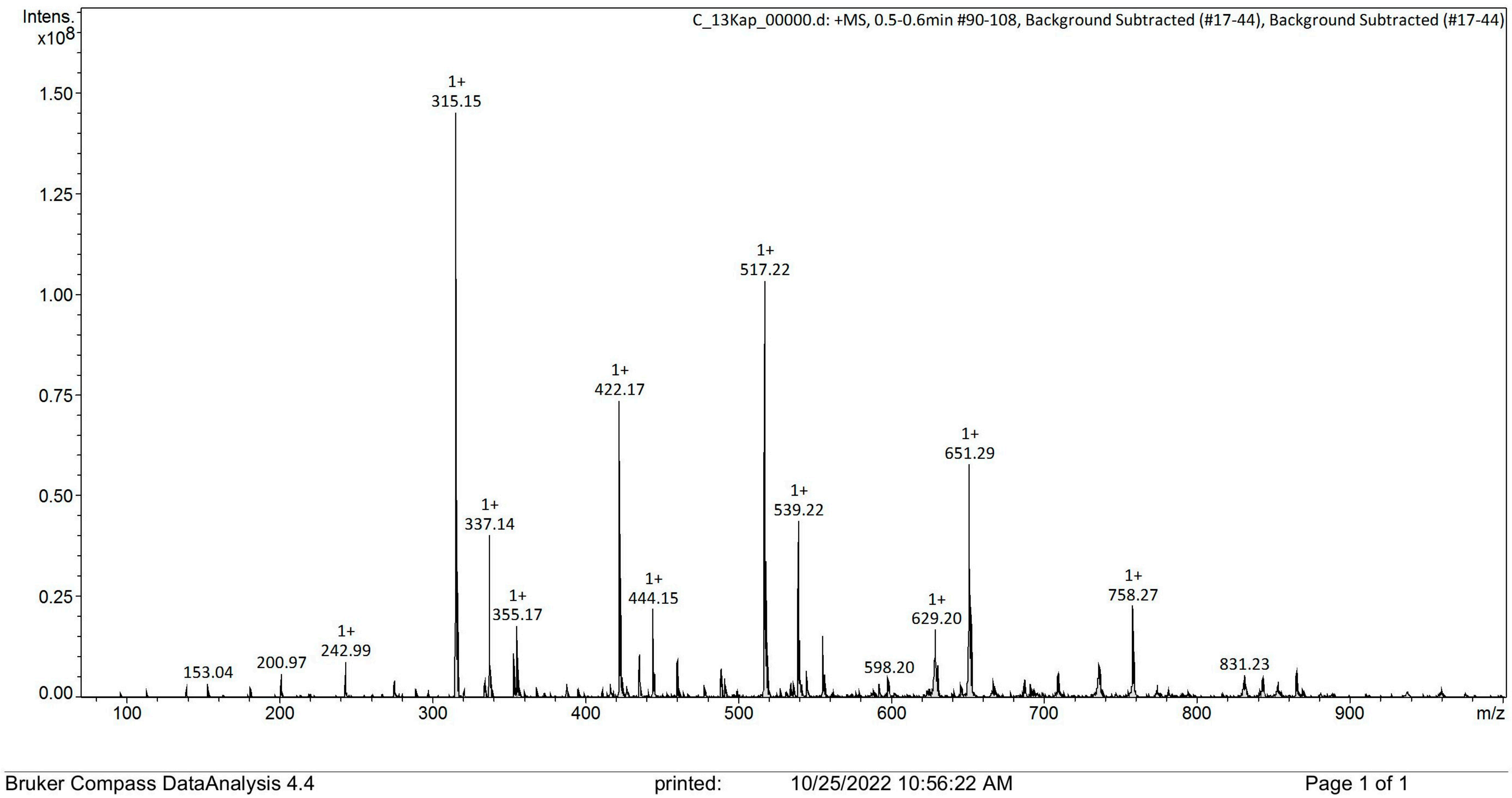

Figure S49. ESI spectrum of compound **3e**

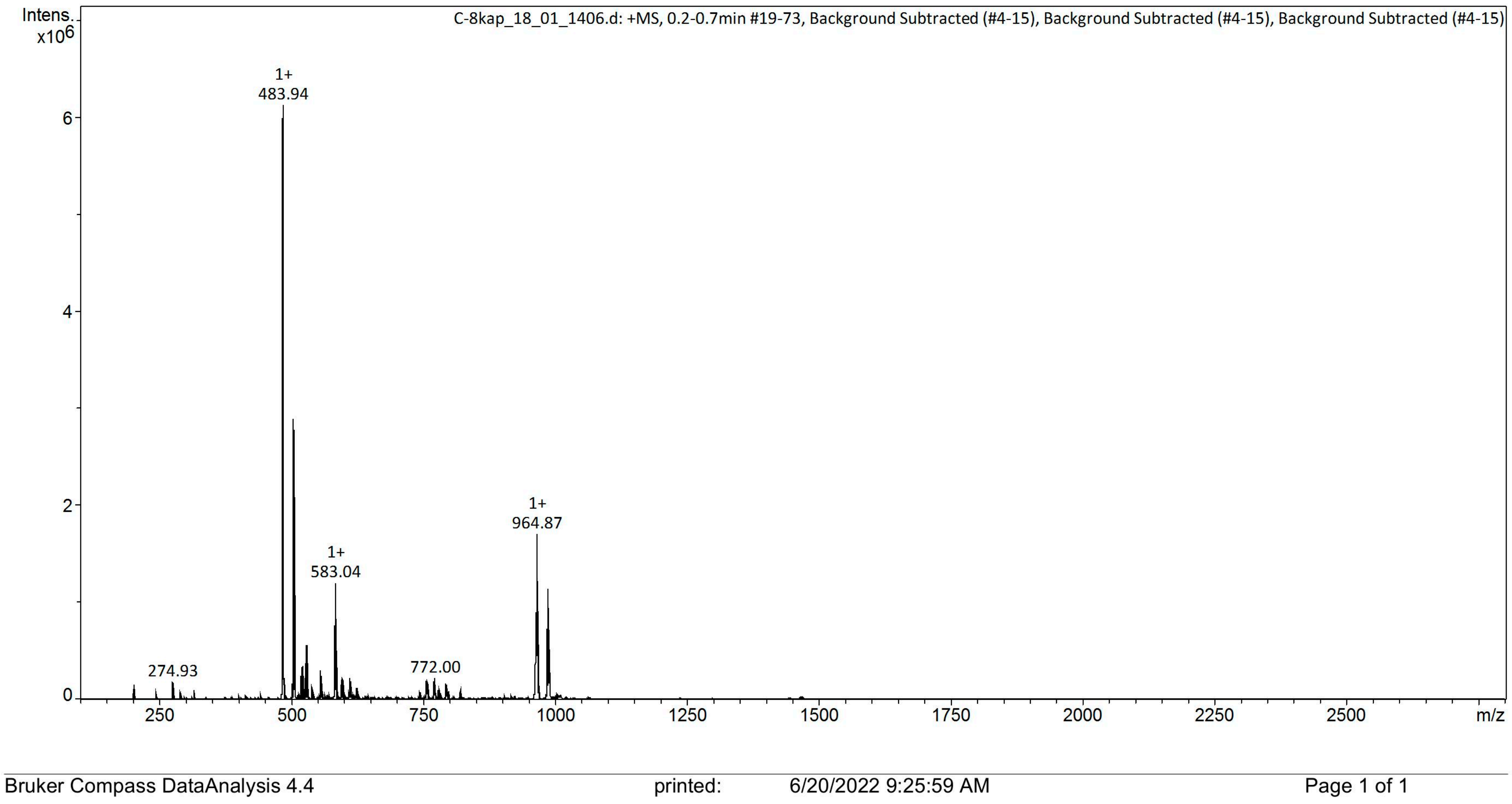

Figure S50. ESI spectrum of compound **3f**

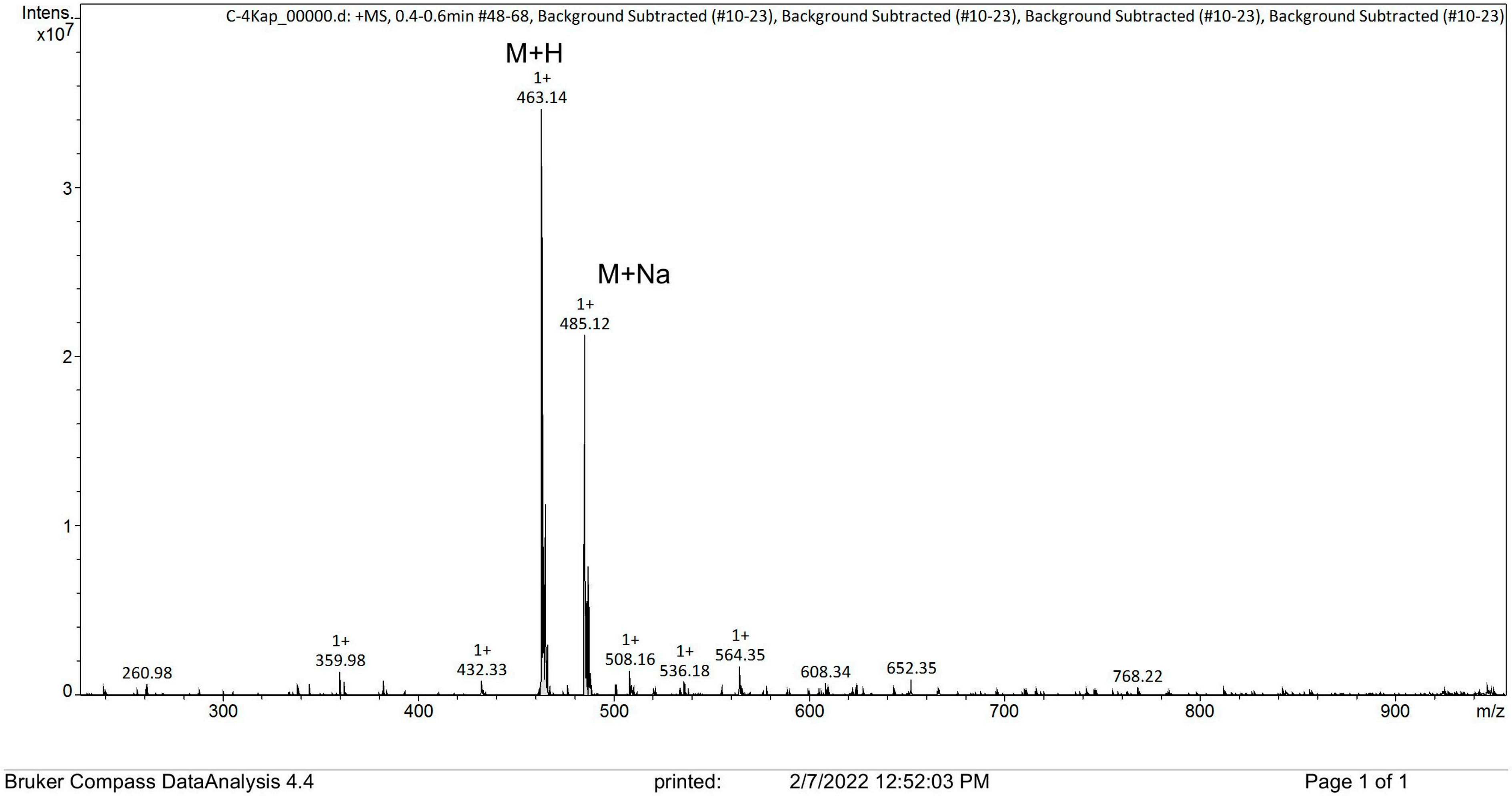

Figure S51. ESI spectrum of compound **5a**

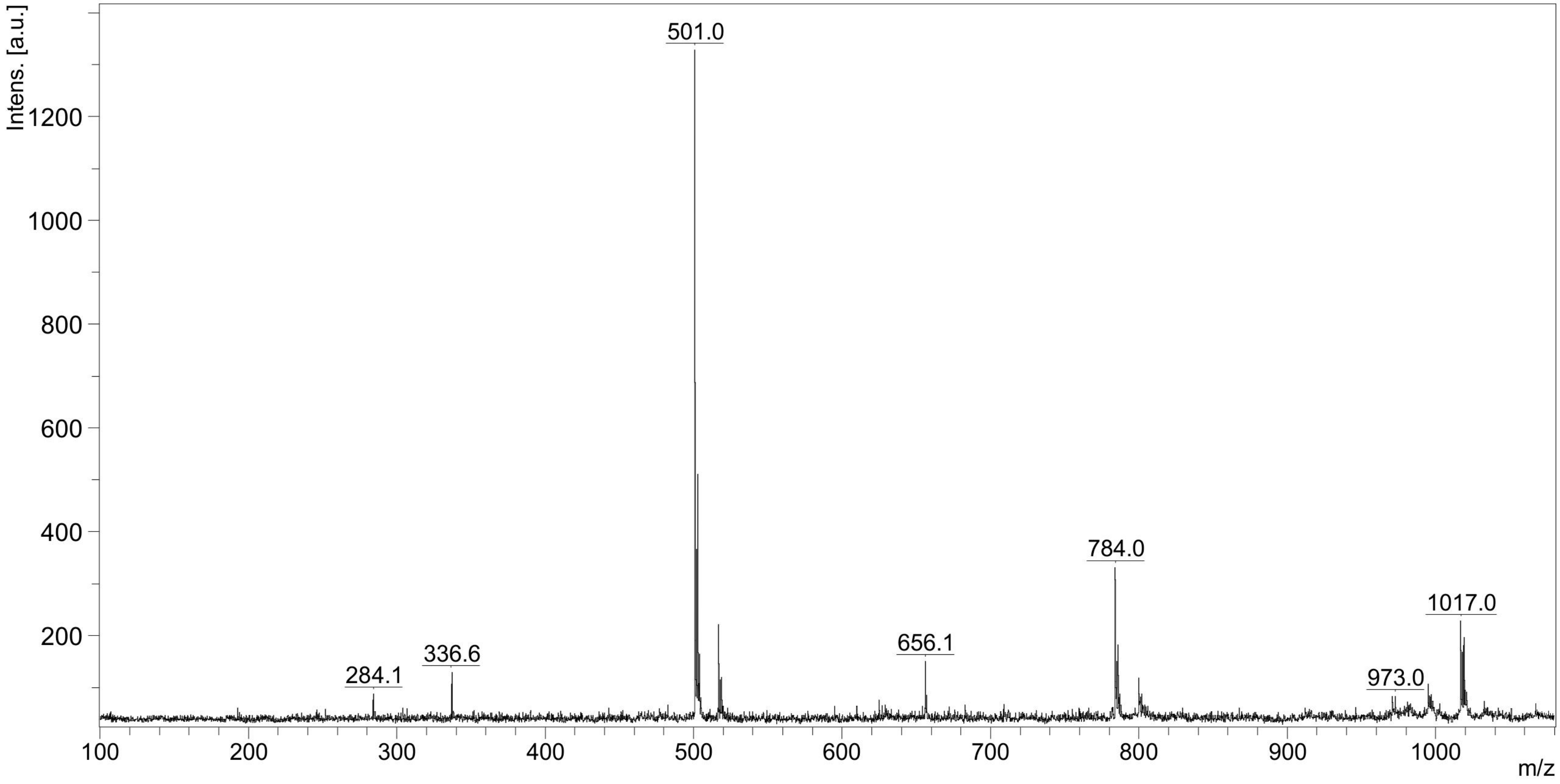

Figure S52. MALDI spectrum of compound **5b**

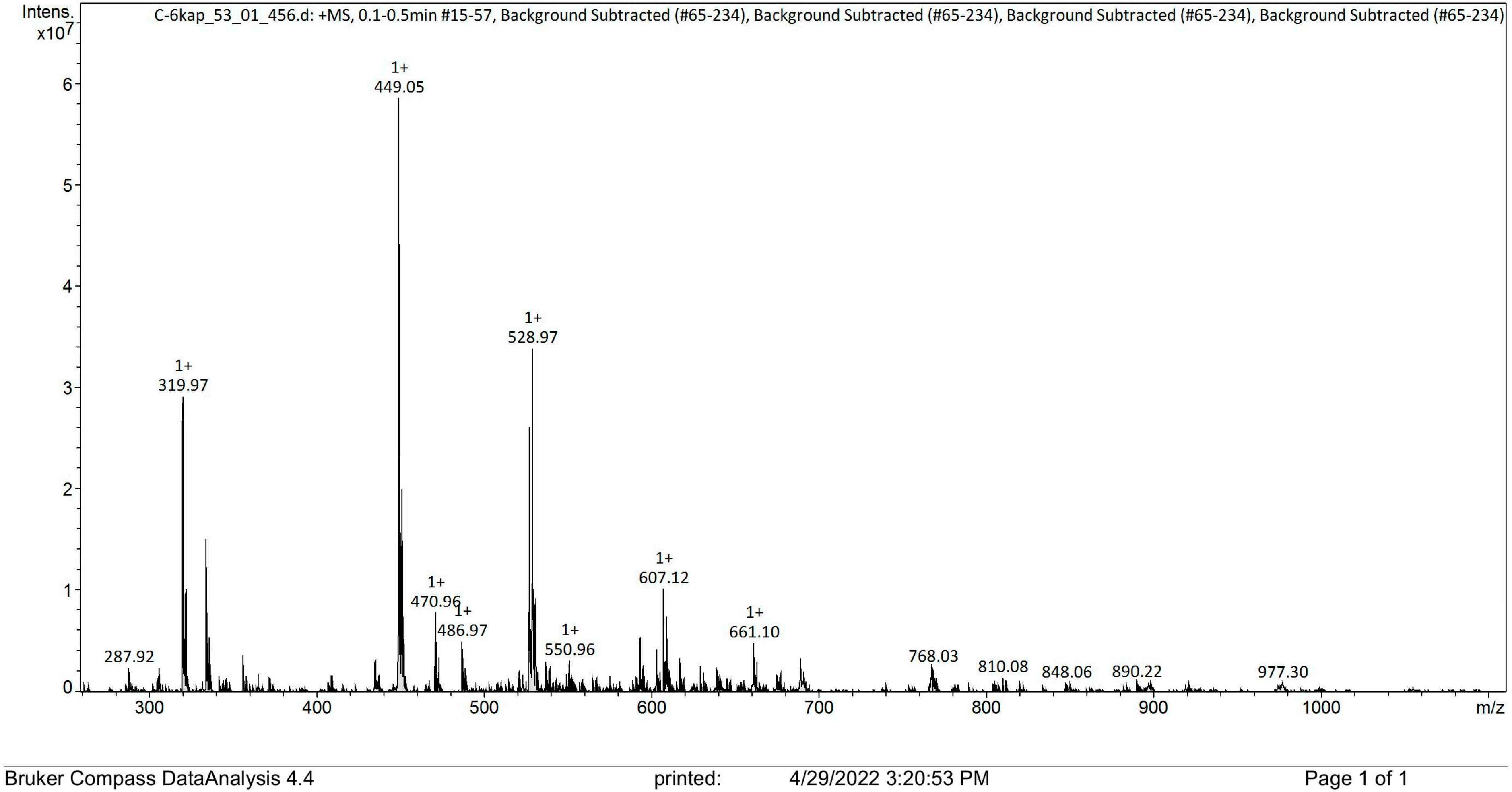

Figure S53. ESI spectrum of compound **5c**

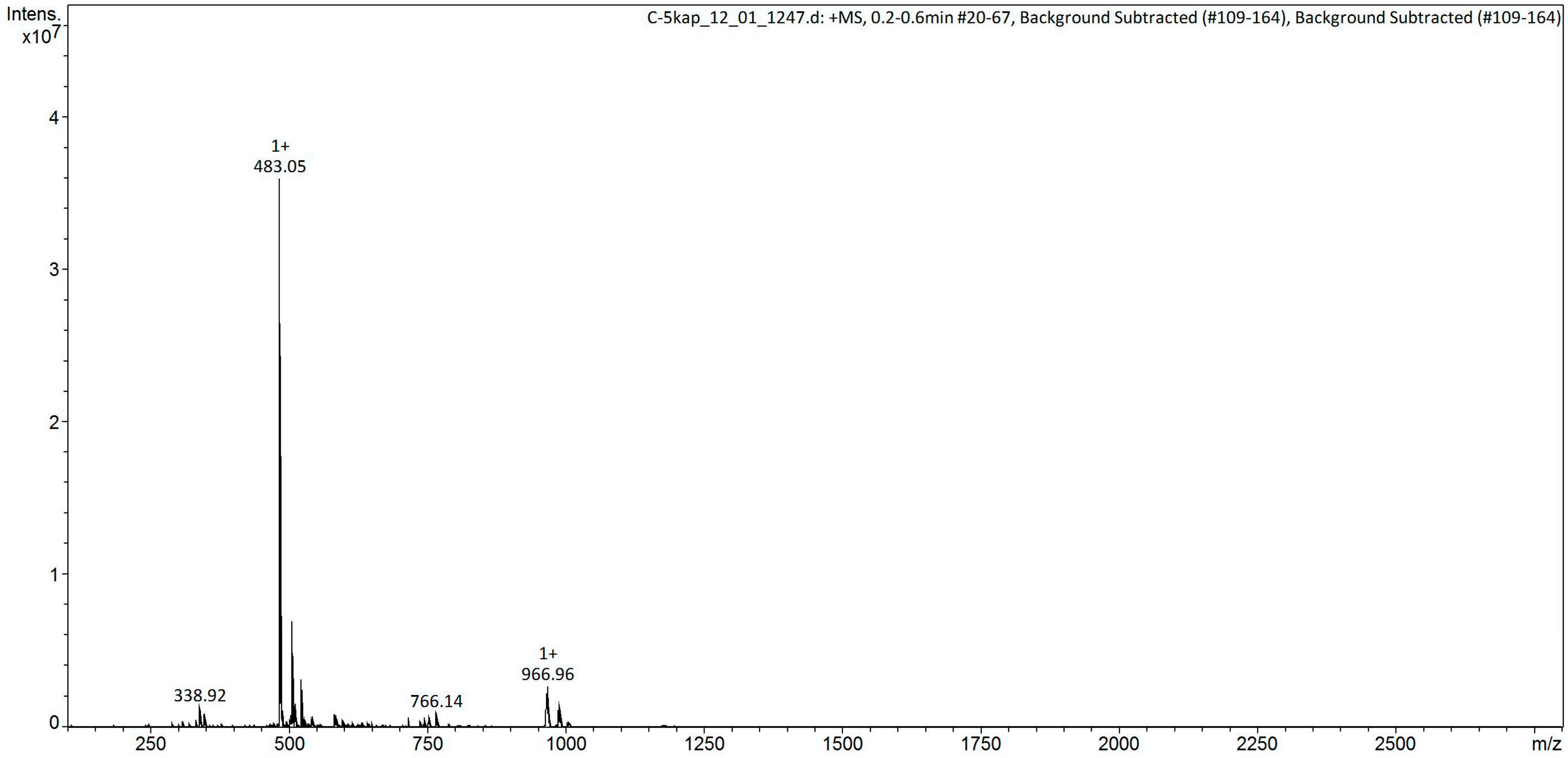

Bruker Compass DataAnalysis 4.4 printed: 6/1/2022 12:21:04 PM Page 1 of 1

Figure S54. ESI spectrum of compound **5d**

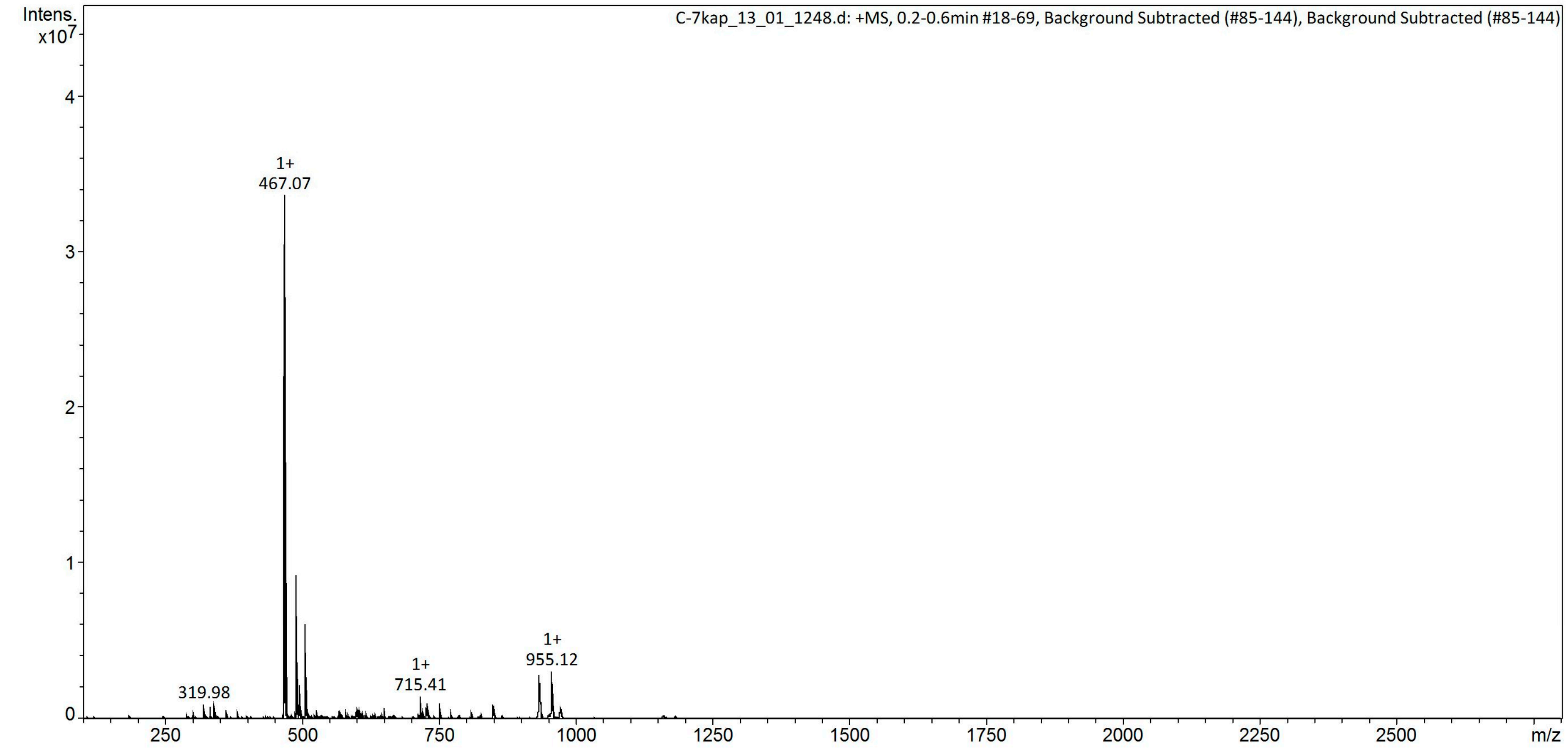

Bruker Compass DataAnalysis 4.4 printed: 6/1/2022 12:21:32 PM Page 1 of 1

Figure S55. ESI spectrum of compound **5e**

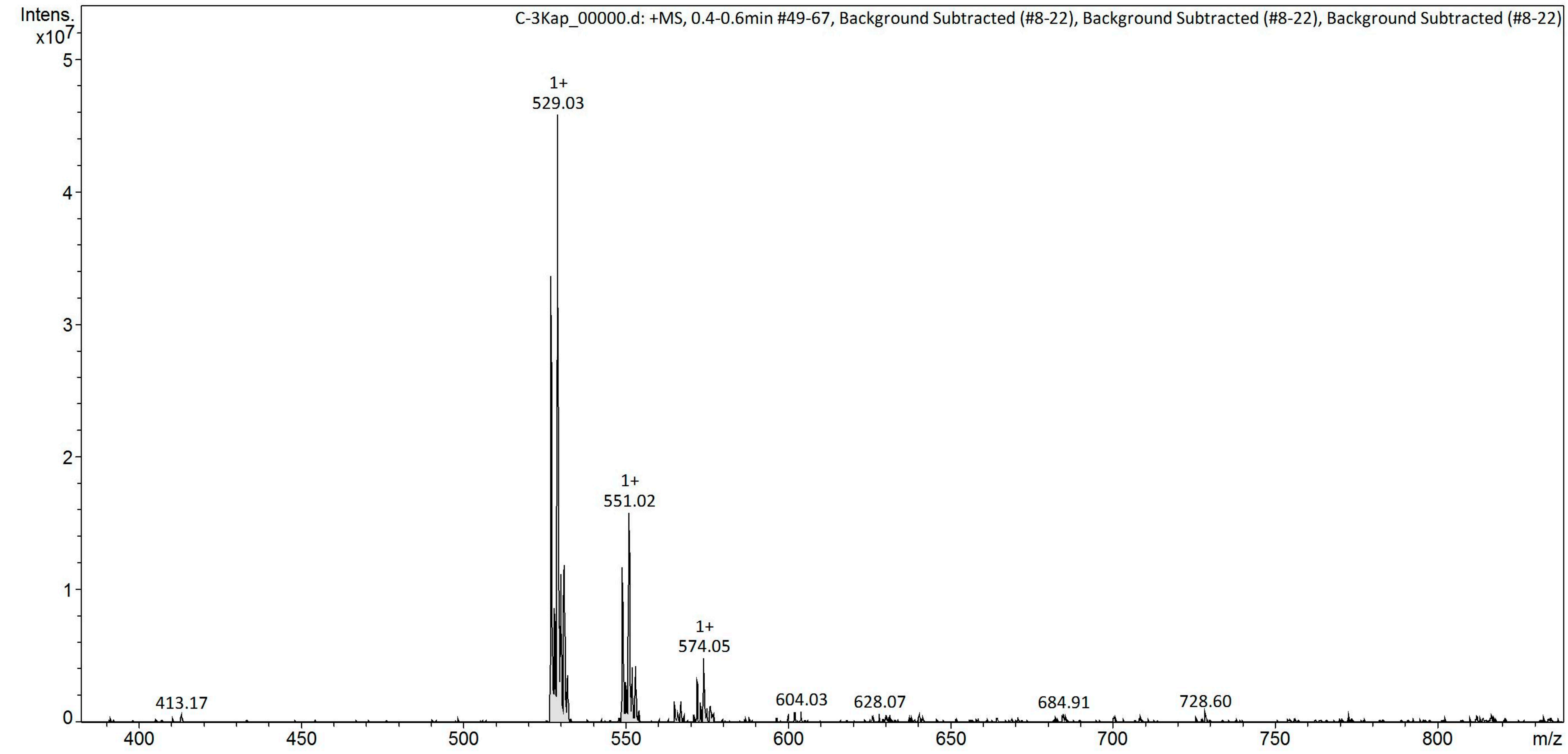

Bruker Compass DataAnalysis 4.4 printed: 2/7/2022 12:48:49 PM Page 1 of 1

Figure S56. ESI spectrum of compound **5f**
